# Unveiling plasmid diversity and functionality in pristine groundwater

**DOI:** 10.1186/s40793-025-00703-8

**Published:** 2025-04-24

**Authors:** Olga María Pérez-Carrascal, Akbar Adjie Pratama, Matthew B. Sullivan, Kirsten Küsel

**Affiliations:** 1 Aquatic Geomicrobiology, Institute of Biodiversity, Friedrich Schiller University Jena, Jena, Germany; 2 Cluster of Excellence Balance of the Microverse, Friedrich Schiller University Jena, Jena, Germany; 3 German Centre for Integrative Biodiversity Research (iDiv) Halle-Jena-Leipzig, Leipzig, Germany; 4 Department of Microbiology, The Ohio State University, Columbus, Ohio USA; 5 Byrd Polar and Climate Research Center, Ohio State University, Columbus, Ohio USA; 6 Center of Microbiome Science, Ohio State University, Columbus, Ohio USA; 7 Department of Civil, Environmental and Geodetic Engineering, Ohio State University, Columbus, Ohio USA

**Keywords:** Plasmids, Groundwater, Microbiome, Metagenomics

## Abstract

**Background:**

Plasmids are key in creating a dynamic reservoir of genetic diversity, yet their impact on Earth’s continental subsurface—an important microbial reservoir—remains unresolved. We analyzed 32 metagenomic samples from six groundwater wells within a hillslope aquifer system to assess the genetic and functional diversity of plasmids and to evaluate the role of these plasmids in horizontal gene transfer (HGT).

**Results:**

Our results revealed 4,609 non-redundant mobile genetic elements (MGEs), with 14% (664) confidently classified as plasmids. These plasmids displayed well-specific populations, with fewer than 15% shared across wells. Plasmids were linked to diverse microbial phyla, including *Pseudomonadota* (42.17%), *Nitrospirota* (3.31%), Candidate Phyla Radiation (CPR) bacteria (2.56%), and *Omnitrophota* (2.11%). The presence of plasmids in the dominant CPR bacteria is significant, as this group remains underexplored in this context. Plasmid composition strongly correlated with well-specific microbial communities, suggesting local selection pressures. Functional analyses highlighted that conjugative plasmids carry genes crucial for metabolic processes, such as cobalamin biosynthesis and hydrocarbon degradation. Importantly, we found no evidence of high confidence emerging antibiotic resistance genes, contrasting with findings from sewage and polluted groundwater.

**Conclusions:**

Overall, our study emphasizes the diversity, composition, and eco-evolutionary role of plasmids in the groundwater microbiome. The absence of known antibiotic resistance genes highlights the need to preserve groundwater in its pristine state to safeguard its unique genetic and functional landscape.

**Supplementary Information:**

The online version contains supplementary material available at 10.1186/s40793-025-00703-8.

## Background

Microbial cells contain mobile vectors that carry unique and adaptative genetic material from one cell to another [1]. This pool of mobile genetic elements (MGEs) is called the cell mobilome [2, 3]. Among these MGEs, plasmids stand out as self-replicating DNA molecules that play a critical role in horizontal gene transfer (HGT) and microbial evolution. Plasmids can be acquired by the host through various cellular mechanisms such as conjugation, transduction, transformation, and vesiduction, i.e. DNA transfer by extracellular vesicles [4]. They are often referred to as part of the accessory genome and as selfish [5]. Plasmids make up a large part of the genetic repertoire in some microbial cells (around 30% of the cell genomic content) [6, 7] and are considered part of the core genome. They co-evolve with host chromosomes, becoming essential for adaptation, stress response and symbiotic associations (e.g., microbe-plant interactions) [8–10]. Plasmids mediate the HGT between mostly phylogenetically closely related cells, although they can successfully circumvent taxonomic boundaries including broader taxonomic ranks such as order, class, and phylum [11]. Adaptive plasmids allow microbes to thrive and increase their ecological niche breadth [5, 12]. In the absence of a selective advantage, plasmids may be lost by microbial cells due to the metabolic burden they impose during replication. Plasmids can also persist across multiple generations within complex microbial communities, even under varying selective forces that should lead to their loss, a situation referred to as the “plasmid paradox“ [5, 13].

Depending on the mechanism of mobilization through cells, plasmids fall into three general categories: conjugative, mobilizable, and non-mobilizable. Conjugative plasmids contain all the necessary machinery for self-transfer, including the mating-pair formation (MPF) system required for conjugation. In contrast, mobilizable plasmids lack the MPF system but can be transferred by hitchhiking on the MPF of co-occurring conjugative plasmids. Non-mobilizable plasmids lack the genes for self-transfer and are moved between cells through alternative mechanisms such as natural transformation, transduction, or membrane vesicles [1, 14].

Plasmids are ubiquitous across nearly all environments on Earth, hosted by a diverse range of microbes, including archaea (at least 4 phyla and 7 classes) and bacteria (at least 45 phyla and 94 classes) [15, 16]. They have also been identified in various eukaryotic cells [17]. In bacteria, plasmids have been mostly associated with the phyla *Pseudomonadota* (formerly *Proteobacteria*), *Bacillota* (formerly *Firmicutes*), *Bacteroidota*, and *Actinobacteriota*. In certain microbial growth forms, such as biofilms, the availability and mobility of plasmids between cells are enhanced due to the high frequency of cell-cell interactions, which are facilitated by the elevated cell density (up to 10^11^ cells/mL), and the structured spatial organization of the community [18–21]. High cell density environments are recognized as hotspots of enhanced HGT, primarily through conjugation, and less likely via transformation and transduction [18, 22, 23]. These environments also foster cooperation and competition among bacteria [24–26]. In fact, most pioneering studies on the plasmidome—the total plasmid content in a given environment—have focused on environments with high cell densities, such as the rumen and the human gut [27–29], as well as human-impacted settings such as wastewater [30]. These studies often emphasize drug resistance genes and frequently associate findings with *Pseudomonadota*. While there is a growing interest in naturally occurring plasmids within aquatic environments such as oceans and groundwater, which typically exhibit lower microbial cell densities [31, 32], the plasmidome composition in these settings remains largely understudied [33].

Groundwater microbiomes constitute a significant fraction Earth´s microbial life, encompassing an estimated 5 × 10^27^ cells globally, which accounts for approximately 10% of the planet´s total microbial biomass [21, 34]. This underscores the pivotal role of groundwater ecosystems in global biogeochemical cycles [35–38]. Despite their importance, our understanding of genetic elements such as plasmids within these environment remains limited, particularly in abundant yet underexplored taxa such as nanoarchaea and the ultra-small Candidate Phyla Radiation (CPR) bacteria [39, 40]. Plasmids are known to play vital roles in microbial adaptation, influencing metabolism, gene flow, and cooperation through shared resources such as public goods. However, their prevalence, diversity, and functional roles in groundwater ecosystems are still poorly understood. These genetic elements may also serve as sensitive indicators of environmental threats, such as anthropogenic pollution. For instance, plasmids from groundwater exposed to metal waste have been found to carry genes critical for mercury detoxification [41–43], highlighting their adaptive significance.

We hypothesize that groundwater microbiomes act as reservoirs for novel and uncharacterized plasmids, harboring genes that are essential for maintaining subsurface biogeochemical processes. Investigating these plasmids at deeper genomic and functional levels is crucial for unraveling their roles in ecosystem dynamics and their potential to address emerging environmental challenges.

In this study, we analyzed a collection of short-read metagenomic sequencing samples (*n* = 32, with data ranging from 16.4 to 22.1 Gbp) from six groundwater wells, using sequential filtration (0.1 μm and 0.2 μm) to capture microbial diversity. This dataset was complemented by a previously published metatranscriptomic dataset (*n* = 17) derived from the same wells, except for H14 [36, 39, 44]. All samples were obtained from a pristine groundwater system within the Hainich Critical Zone Exploratory (Thuringia, Germany), where CPR bacteria dominate the microbial community, comprising 23–79% of the total microbiome [36, 39, 44]. By integrating short-read metagenomic data with multiple plasmid prediction tools, we identified 4,609 MGEs, 664 of which were confidently classified as plasmids. These MGEs were taxonomically linked to microbial hosts using both sequence alignment tools (e.g., BLAST) and a CRISPR-based detection approach [45, 46]. While this combination of tools provides valuable insights, the inherent complexity of plasmid genomes, coupled with the limitations of short-read sequencing and current prediction algorithms, can constrain the completeness of plasmid recovery and the accuracy of host assignments. These challenges underscore the importance of advancing methodological approaches—such as the integration of long-read sequencing technologies, optimized plasmid DNA extraction protocols, and improved host-mapping techniques—to more comprehensively resolve the diversity and ecological roles of MGEs [47, 48]. We aimed to (i) explore the diversity of mobilome, with a particular focus on plasmids, and their genetic and functional composition, (ii) characterize the potential host of plasmids, shedding light on their distribution across phylogenetic groups, (iii) investigate correlations between microbial composition and the plasmid pool across sampling sites, and (iv) reveal the role of plasmids in mediating the transfer of genetic information, including public goods and antibiotic resistance genes. This work provides valuable insights into the genetic landscape of groundwater microbiomes, emphasizing the ecological and evolutionary significance of plasmids in these systems.

## Methods

### Sampling site description and metagenomic data collection

Mobile genetic elements (MGEs), specifically plasmids, were characterized in metagenomes (*n* = 32) obtained from groundwater collected along six selected wells distributed in a hillslope fractured carbonate aquifer system of the Hainich Critical Zone Exploratory (CZE) located in Thuringia, Germany, in November 2018 and January 2019. The present study focuses on samples obtained from six out of 10 groundwater wells (H14, H32, H41, H43, H51, H52), which represent a diverse range of environmental conditions, including oxic and anoxic zones, as well as limestone-dominated and mudstone-dominated aquifer assemblages [49, 50]. Wells H14, H32, H41, and H51 exhibit oxic conditions, whereas wells H43 and H52 have suboxic or anoxic conditions. More site characteristics and sampling details are described elsewhere [36, 39]. In short, between 50 and 100 L of groundwater were sequentially filtered in January 2019 from each well through 0.2 μm filters and 0.1 μm filters per triplicate to capture the small sized bacteria and archaea, except for H32, which had only one replicate of the 0.2 μm fraction. A high particle load in the H32 sample caused early filter clogging, preventing further filtration. Thus, we included an additional sample from the 0.2 μm fraction, collected during the November 2018 sampling campaign, for downstream analyses [36]. DNA extractions were performed using a phenol-chloroform method. A total of 32 metagenomes were sequenced using Illumina NextSeq 500 system and paired-end library (2 × 150 bp) [36, 39].

### Metagenome and metatranscriptomic data characteristics

The metagenome sequences were quality-filtered as described in two previous studies (European Nucleotide Archive (ENA) projects: PRJEB36505 and PRJEB36523) [36, 39]. The quality-filtered reads were used for metagenomic assemblies with metaSPAdes (version 3.12) [51] and subsequent analyses. An additional metatranscriptome dataset (*n* = 17) collected in August and November 2015 published under the ENA project PRJEB28738 was used to validate the presence of genes in MGEs under transcription [44]. Groundwater samples were collected from the same wells, except for H14, which was not included in this earlier sampling campaign.

### Prediction of MGEs, particularly plasmids

To investigate MGEs classified mainly as plasmids, we used three approaches developed for plasmid identification. First, we used the SCAPP (Sequence Contents-Aware Plasmid Peeler) (version 0.1.1) [52] tool with metaSPAdes assembly graph and used as -k/–max_kmer value 55, keeping the other parameters as default [51]. The alignment of the reads to the assembly graph was performed using SAMtools (version 1.15.1) [53]. The SCAPP approach was successfully applied to all the wells, however, due to the high number of nodes per assembly, SCAPP was not applied to several sample replicates (7 out of 32). The second approach was to use PlasX [54], a tool that uses machine learning to classify plasmids in metagenome-assembled sequences based on genetic architecture. Sequences with a score greater than or equal to 0.90 were considered as high confidence plasmids. The preprocessed fasta files used as input in PlasX were processed using Anvi’o (version 7.1) [55]. Finally, the third approach consisted of building a co-assembly of the metagenome replicas using MEGAHIT (k-mer = 77) (version 1.2.9) [56] and the program DomCycle with default parameters [57]. The MEGAHIT co-assemblies in fasta format were converted to fastg using the contig2fastg function implemented in MEGAHIT [56]. For each co-assembly, the read mapping of their corresponding reads was performed using BWA-MEM (version 0.7.17) with default parameters [58]. DomCycle was executed with default parameters and matching co-assembly k-mer.

For each groundwater sample, the contigs recovered using the three approaches were merged and dereplicated with the program MobMess [54], testing complete and fragmented sequences, using values of similarity and coverage of the smaller plasmid ≥ 90%. This program selects one representative sequence per cluster with the highest global sequence identity. Clustering up to 95% similarity gave similar results (differed by only for 2 clusters). After dereplication with MobMess, the MGEs from all the samples were pooled, dereplicated, and used for further comparative genomic analyses. Additionally, we used dRep [59] 99% identity for secondary clustering, and approximately 95% of the dereplicated sequences identified with MobMess were also identified with dRep. Non-redundant MGE-DNA contig sequences are available on https://zenodo.org/records/14500014.

### Reclassifying MGEs, mainly plasmids using protein sequence annotation and virus prediction tools

Although the prediction of MGEs was mostly done with bioinformatics tools designed to identify plasmids, it is important to note that some tools, such as SCAPP, can identify circular MGEs that are distinct from plasmids [43]. So, additional steps, such as protein sequence annotation, were required to infer whether plasmids were classified as other MGEs. Thus, we obtain a more conservative prediction of the plasmid sequences.

First, MGEs were classified into three types: plasmids, phages, and other mobile elements based on the protein sequence annotation using Pfam, COGs, KEGG, and eggNOG databases [60–63]. One MGE was defined as a plasmid, phage, and unassigned MGE (uMGE) based on the protein terms defined by Shalon et al., 2022 and Yu et al., 2024 [54, 57].

Briefly, if the MGEs contain genes annotated as plasmid, conjugation, mobilization (*mobA*, *mobB*, *mobC*, *mobD*, and *mobE*), and partition system (*parA* and *parB*), they were classified as plasmids. If the MGEs contain at least one functional protein annotation with the following terms: capsid, phage, tail, head, tape, antitermination, virus, bacteriophage, sipho, baseplate, T4-like, and myovir, they were classified as phages. MGEs without match with the terms described above but with gene annotations such as transposases, transposons, toxin-antitoxin (TA) systems, excision, integrase, relaxase, recombination, segregation, extrachromosomal, mobilization, and partitioning were classified as unassigned MGEs (uMGEs).

To exclude the presence of phages that could be misclassified as plasmids, we additionally used the tools VirSorter2 (version 2.0.9) [64], VIBRANT(version 1.2.1) [65] and DeepVirFinder (version 1.0) [66]. VirSorter2 retained the viral sequences with a quality score of 0.9 (version 2.0.9). DeepVirFinder (version 1.0) only considered those sequences with scores equal to 0.9 or more and qvalues of 0.05 or less. The program geNomad (version 1.5.1) [67] with conservative parameters was used to complete the classification of plasmids based on the conjugative genes and viral sequences. Altogether, the results from VirSorter2,, DeepVirFinder, and geNomad were considered to classify viral sequences in our MGE dataset. Finally, using CONJscan database [68] as is implemented in geNomad and MacSyFinder (version 2.0), we identified plasmids containing relaxase mobilization (MOB) genes [69] (Supplementary Table S1). A workflow summarizing the methods described above is shown in Supplementary Figure S1.

### Inferring the MGEs novelty

The similarity of the plasmid to other plasmids in public databases was checked using blastn (≥ 90% coverage, 60% identity, and e-value < 1e-5; as implemented in the default parameters of the PLSDB search tool (version 2.12.0) [70, 71]) and Mash (minimum identity = 99) (version 2.2) against the PLSDB database version 2020_06_23 [72], which contains 34,513 reference plasmids. The best matches were selected in both blastn and Mash outputs.

### Inferring the MGEs potential host MAGs taxonomy and associations

We used a database of metagenome-assembled genomes (MAGs) derived of two previous studies (*n* = 2,589) (genome completeness ≥ 50% and contamination ≤ 10%) [36, 39] (Supplementary Table S2). The MAGs were taxonomically annotated using the function ‘classify’ implemented in GT-DBTk (version 2.1.0; r207) [73]. Then, the MAGs were classified into three categories based on the taxonomic annotation: bacteria (non-CPR), archaea, and CPR. To increase the number of possible MGEs-MAGs associations, the non-dereplicated MAGs were also included in the analysis. MAG completeness and redundancy were assessed using CheckM (version 1.2.0) [74] (Supplementary Table S2). A set of 43 markers proposed by Brown et al., 2015 was used to estimate the completeness and redundancy of the CPR MAGs [75].

To infer the association between MGEs and MAGs we used two approaches. First, we used the Mash screen algorithm (version 2.2) (minimum identity = 99 and shared hashes of at least 800/1,000) [46]. Briefly, a database with the MGEs (1,000 bp length) was created using the sketch function in Mash (k-mer = 21 and sketch size = 1,000). This database was subsequently used to estimate the number of shared hashes between the MGEs and the individual MAGs. Second, we did host assignment for the non-redundant plasmids based on the iPHoP (version 1.3.3) [45] framework with the default parameters and the database “Aug_2023_pub” and MAG groundwater custom database (*n* = 2,589). Only the top hits were considered in this approach. We then merged Mash and iPHoP results.

A concatenated protein alignment generated with GTDB-Tk for bacteria and a separate alignment for archaea (refined and dereplicated MAGs only) were used to infer phylogenetic trees. The maximum likelihood trees were estimated (bacteria and archaea) using IQ-TREE tool (version 2.2.0.3) [76] and the protein substitution model Whelan and Goldman (WAG) [77]. The phylogenies were visualized together with the MGEs-MAGs associations using iTOL (version 6.8) [78].

An additional way to identify the host chromosome of our MGEs was using the CRISPRspacers present in the MAGs. CRISPR-Cas arrays in groundwater MAGs (*n* = 2,589) were identified using CRISPRCasFinder (version 4.2.30) [79]. The CRISPR-Cas arrays with levels of evidence 3 and 4 were used to extract the CRISPRspacers. The spacers corresponding to each array with a length greater than 25 bp were aligned against the MGEs database using blastn-short [70]. Only the hits with alignment coverage 100%, ≤ 2 mismatches, and sequence identity ≥ 90% were kept and considered as protospacer-to-spacer matches as described previously by Hwang et al. [80]. The spacers in the CRISPR-Cas arrays were used to predict the potential MGE-MAG connections.

### MGEs and MAGs community composition across the Hainich transect based on coverage depth

To estimate the coverage depth of each MGE, the metagenomic reads for each of our 32 metagenomes were mapped using minimap2 (version 2.24) [81] as implemented in CoverM (version 0.6.1) (https://github.com/wwood/CoverM). The coverage depth was based on the MetaBAT method (adjusted coverage) [82](Parameters:–min-read-percent-identity 0.95–trim-min 0.05–trim-max 0.95 --min-covered-fraction 0). MGE coverages were normalized across samples using a scaling factor calculated based on the number of RNA polymerase B (*rpoB*) genes in the quality-filtered reads, as was previously described (Supplementary Table S3) [36, 39].

MGEs with coverage depth greater than 1 were considered to be present in the replicates. MGEs were separated into three types: plasmids, phages, and uMGEs (Supplementary Table S4). We determined the presence/absence of MGEs across the replicates, samples, and filter fractions based on the coverage. Furthermore, we identified core MGEs that were present in all wells, and shared within and between filter fractions.

A matrix of coverages was generated for each MGE type and imported in R (version 4.2.2) [83, 84] to estimate the Jaccard distances and Bray-Curtis dissimilarities using the package vegan (version 2.6-4) [85]. These measurements were used to assess the MGE community composition and perform non-metric multidimensional scaling (NMDS) analysis. To evaluate the effect of the site on MGE composition, we used the permutational multivariate analysis of variance (PERMANOVA) test based on the adonis2 function with the parameter by margin.

Likewise, the normalized mean coverages of MAGs were estimated and used for similar analyses. Based on the taxonomy assigned by GTDB-Tk, MAGs were divided into three groups: non-CPR bacteria (*n* = 368), CPR bacteria (*n* = 542), and archaea (*n* = 164).

To test the linkage between MGEs community composition and MAGs community composition, we performed Procrustes and Mantel analyses in R with the package vegan (version 2.6-4) [85]. The coverage matrices were transformed to Bray-Curtis distances and compared using the protest() function with 9,999 permutations. In addition, we evaluated the correspondence between MGEs and MAGs composition using a Mantel test with 9,999 permutations in R.

### Microbe-MGE interactions and network visualization

Sequence coverage depth matrices were generated for each MGE type (plasmids, phages, and uMGEs). Individual MGEs with a total coverage of less than 50X over the entire metagenome sample were filtered out. MGEs with coverage depth of less than 2X per metagenome were also filtered out. In addition, MGEs detected in fewer than 25% of metagenomes were excluded. This allowed us to consider only the MGEs present in at least two different samples or exclude rare MGEs. Coverage matrices for non-CPR bacteria, CPR, and archaea were generated using the same parameters.

Microbe-MGE interaction network analyses were performed using SPIEC-EASI (Sparse Inverse Covariance estimation for Ecological Association Inference) [86] and the neighborhood selection framework (also known as MB method) (version 1.1.2) as implemented in NetCoMi (version 1.1.0) [87]. SPIEC-EASI allowed the estimation of conditional independence between MGEs and the host MAGs. MGEs and MAGs were conditionally independent if their abundances were independent giving the abundances of all the others in the network.

### Metabolic functions of MGEs

Genes of MGEs were functionally annotated using the tool DRAM (version 1.4.6) [88], which includes annotations based on the MEROPS peptidase [89], KOfam [90], Pfam [60], and dbCAN/CAZYmes databases [91]. The MGEs were merged according to sample repeats and divided into the three MGE types (plasmids, phages, and uMGEs). Gene prediction was performed using Prodigal (version 2.6.3) [92].

Presence/absence matrices were generated for all the MGEs, plasmids, phages, and uMGEs based on functional annotations. Hierarchical clustering of samples was performed using the hclust function implemented in the pheatmap R package (version 1.0.12) [93].

To know the functional contribution of the MGEs per type to the microbial community, the relative abundances of each ) functional category based on the Clusters of Orthologous Genes (COG) were estimated for each MGE type. We used eggNOG mapper (version 2.1.9; database version 5.0.2) [63] to functionally annotate genes using the COG20 database [94, 95]. To assess the presence of antibiotic resistance genes (ARGs), we annotate MGE contigs using the Resistance Gene Identifier (RGI, version 6.0.2) in the Comprehensive Antibiotic Resistance Database (CARD, version 3.2.7) with default parameters. Further, gene matches with ARGs were organized into three categories: perfect, strict, and loose as previously described [96]. In addition, we searched for ARGs in MGEs using the NCBI AMRfinder tool (version 3.11.18) [97].

To assess the linkage between MGE coverage depth and environmental parameters (Supplementary Table S4), we calculated Spearman’s rank correlation coefficient using the R cor function. The statistical significance of the correlations was determined through a permutation test with 999 iterations. We then compared the functional annotations of genes in both MGEs with and without strong correlations, identifying the functions that were uniquely associated with the MGEs showing strong correlations.

### Gene expression in MGEs

The quality-filtered metatranscriptomic reads (*n* = 17) were used as input in Kallisto (version 0.46.2) [98] to estimate the number of transcripts per gene in the MGEs. The mean of number of transcripts per million (TPM) was calculated based on the number of samples per well (H41, H43, H51, H52). For well H32, where only one sample was available, the mean was not calculated. The TPMs were normalized using a scaling factor based on the *rpoB* gene as was done for the DNA samples, and then expressed in log2 scale and used as input to visualize the gene expression in specific plasmids of interest (i.e., cobalamin and mercury resistance plasmids).

## Results

### Mobile genetic elements (MGEs) exist at all wells in the Hainich CZE

**Fig. 1:**
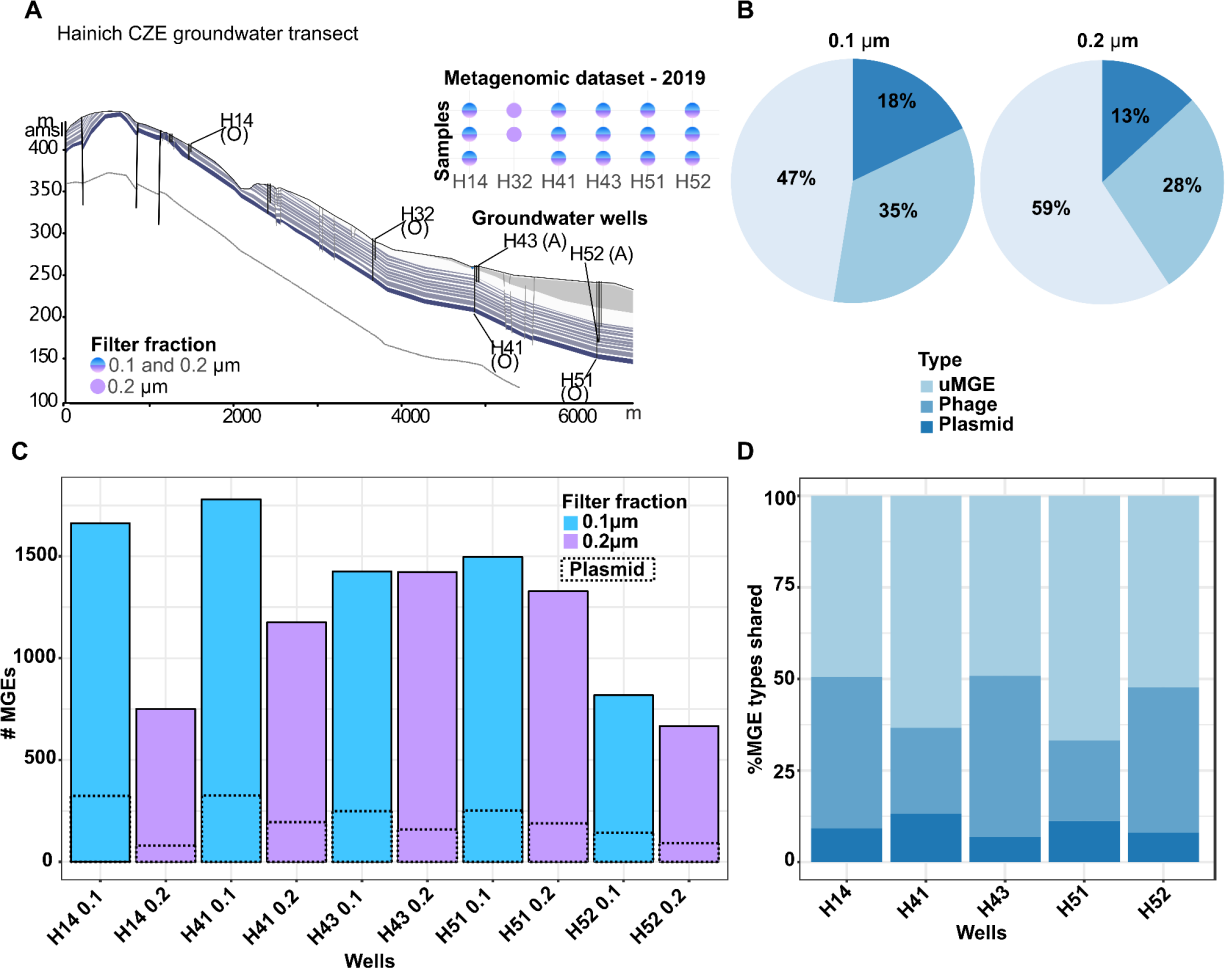
**(A)** Groundwater sampling was conducted at wells representing both oxic (O) and anoxic (A) conditions along the Hainich Critical Zone Exploratory (CZE) transect. Groundwater samples were sequentially filtered through 0.2 μm and 0.1 μm filters, with each fraction being used for metagenomic sequencing. **(B)** Proportions of MGE types in the two filtration fractions. **(C)** MGEs were detected in all groundwater wells. **(D)** Percentage of MGE types common to both 0.1 μm and 0.2 μm filter fractions for each well, calculated based on MGEs detected in both fractions. The well H32 was excluded from the analysis due to the absence of a 0.1 μm filter fraction

We identified a total of 4,609 dereplicate MGE sequences across the six selected groundwater wells of the Hainich CZE that are most characteristic of the spatial differentiation of groundwater microbiomes along the hillslope, with N-compounds and dissolved oxygen as the major determinants [99] (Fig. 1A; Supplementary Table S1). The MGEs were predicted using three plasmid prediction tools—PlasX, SCAPP, and DomCycle—on bulk metagenomic data obtained from two groundwater filter fractions. By utilizing a sequential filtration approach through 0.2 μm and 0.1 μm filters, we ensured that smaller microorganisms, which may play critical roles in groundwater ecology and biogeochemical processes, were not overlooked. Next, we performed dereplication using the MobMess algorithm, applying thresholds of ≥ 90% sequence identity and ≥ 90% coverage. To further characterize the non-redundant MGEs identified, we employed viral prediction tools and protein sequence annotation associated with plasmids. These analyses categorized the MGEs into three distinct types: *i*) plasmids (*n* = 664; 14.41%), of which 119 (17.92%) contain relaxase genes, indicating their potential for mobilization, *ii*) phages (*n* = 1,549; 33.61%), and *iii*) unassigned MGEs (uMGE), encompassing those with ambiguous classification or the presence of transposase and insertion sequences) (*n* = 2,396; 51.98%). This dataset not only provides a comprehensive overview of plasmids but also offers exciting opportunities to investigate other MGEs, such as phages and uMGEs, expanding our understanding of their ecological roles and evolutionary dynamics in groundwater ecosystems.

The predicted MGEs ranged in size from 1,000 bp to 270,036 bp, with a mean length of 8,987 bp. Plasmid sequence lengths were normally distributed with a median size of 4,100.5 bp (Supplementary Figure S2). On average, predicted plasmid sequences account for 0.71% (Standard deviation = 0.58%) of the total sequence length per metagenomic assembly. The distribution of MGE types across filter fractions showed that uMGEs, which were enriched in transposase sequences, were the most abundant, comprising 47–59% of MGEs in both filter fractions. In contrast, plasmids showed the lowest abundance, ranging from 13 to 18% (Fig. 1B). Across all filter fractions and sampling sites, we identified approximately 666 to 1,780 MGEs per sample, depending on the filter fraction and well location (Fig. 1C).

We additionally identified the MGEs that were shared across all wells, within the same filter fraction, and between different filter fractions from the same well. These shared MGEs were categorized as “core MGEs”. The highest proportion of core MGEs was found across both filter fractions within the same groundwater well, with an average of 13.92% (≈ 641 out of 4,609), indicating a reduced overlap between the filter fractions. Of these, 9.73% were plasmids, 34.08% were phages, and 56.19% were uMGEs (Fig. 1D). When considering only the MGEs present in either filter fraction within a sample (mean = 2,505 MGEs), the proportion of core shared sequences averaged 25.70%. In terms of filter fraction-specific shared MGEs, samples from the 0.1 μm filter fraction contained a higher proportion of core MGEs (268 out of 4,609; 5.82%, with 97 being plasmids (97 out of 664 plasmids;14.61%)) compared to the 0.2 μm fraction (26 out of 4,609; 0.56%, with five being plasmids). Notably, there was a small proportion of core MGEs (14 out of 4,609; 0.30%) that were present in all samples, regardless of well or filter size, one of which was identified as a plasmid. Thus, the use of two filter fractions facilitated the exploration of greater diversity within the overall groundwater MGE pool, revealing distinct compositions across different size fractions.

### Groundwater: a reservoir of plasmids linked to diverse and abundant microbial taxa

Next, we explored the potential association between MGEs and their microbial hosts. We found that 37.51% of the MGEs (1,729 out of 4,609) were associated with a host. This group included 59.64% of plasmids (396 out of 664), 20.27% of phages (314 out of 1,549), and 42.53% of unassigned MGEs (uMGEs) (1,019 out of 2,396; Supplementary Table S5). To predict these MGE-host associations, we used two complementary approaches. First, we predicted host associations based on shared k-mer sequences between the MGEs and the MAGs (*n* = 2,589) recovered from the same groundwater metagenomic dataset. Additionally, we applied the integrated phage-host prediction tool (iPHoP [45]), which combines a comprehensive genomic database (including representative genomes in the GTDB [100], the genomes from Earth’s Microbiomes and IMG isolate databases [101]) alongside our custom groundwater MAG database. By incorporating multiple databases and prediction methods, we aimed to enhance potential host predictions, partially addressing the limitations of MGE binning in short-read metagenomic data [102]. Although this approach does not fully resolve the plasmid binning issue, the use of long-read sequencing in future studies may significantly improve the host predictions.

**Fig. 2:**
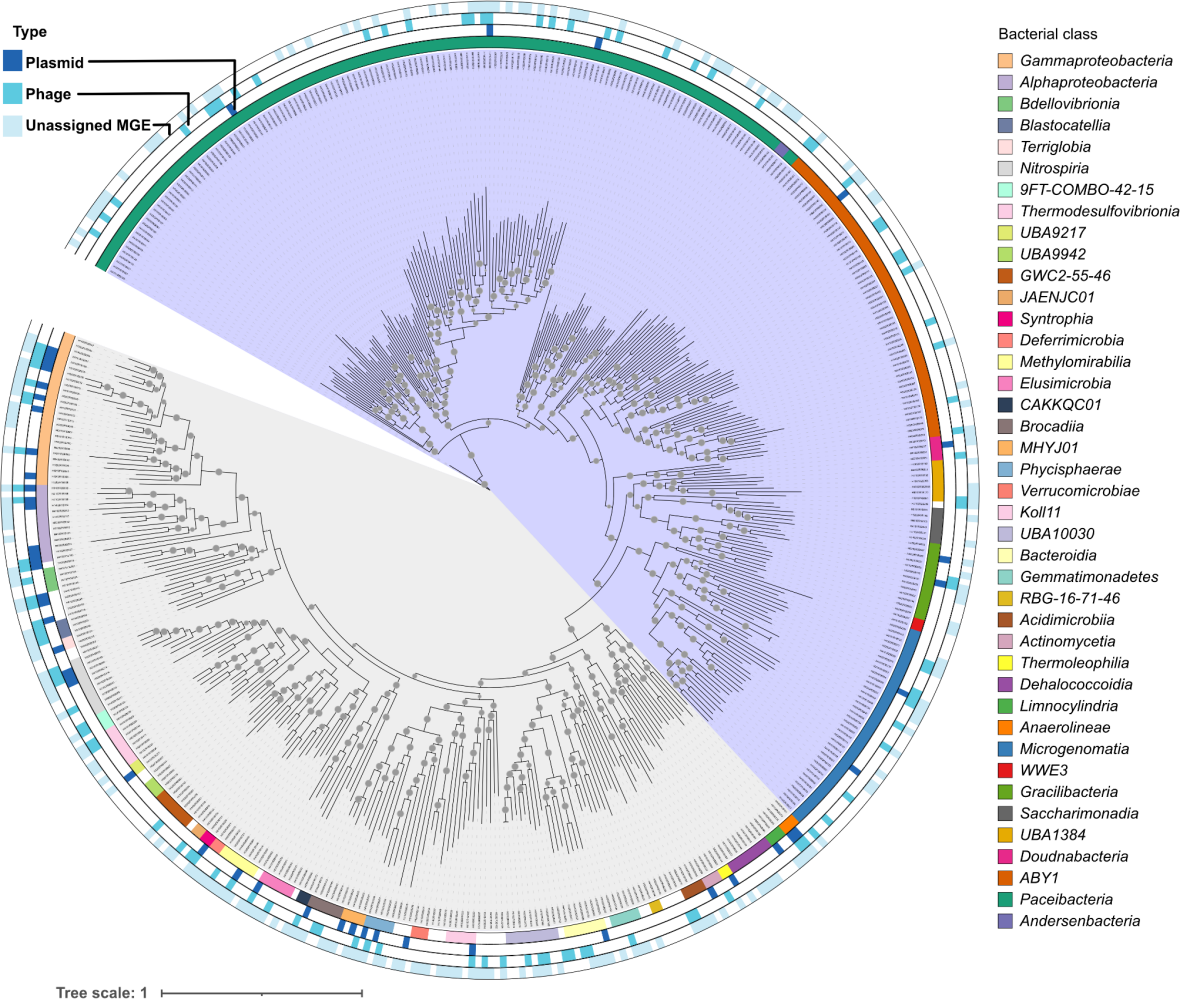
MGEs are associated with multiple microbial lineages in groundwater. The phylogenetic tree was constructed using a protein concatenated alignment of refined MAGs generated with the GTDB-Tk tool. Non-CPR and CPR bacteria are highlighted in gray and purple, respectively. The innermost circle represents bacterial classification by class. The three outermost circles show the presence/absence of MGE associations across groundwater reference and non-redundant MAGs. MGE associations in archaea are shown in Supplementary Figure S3

**Fig. 3:**
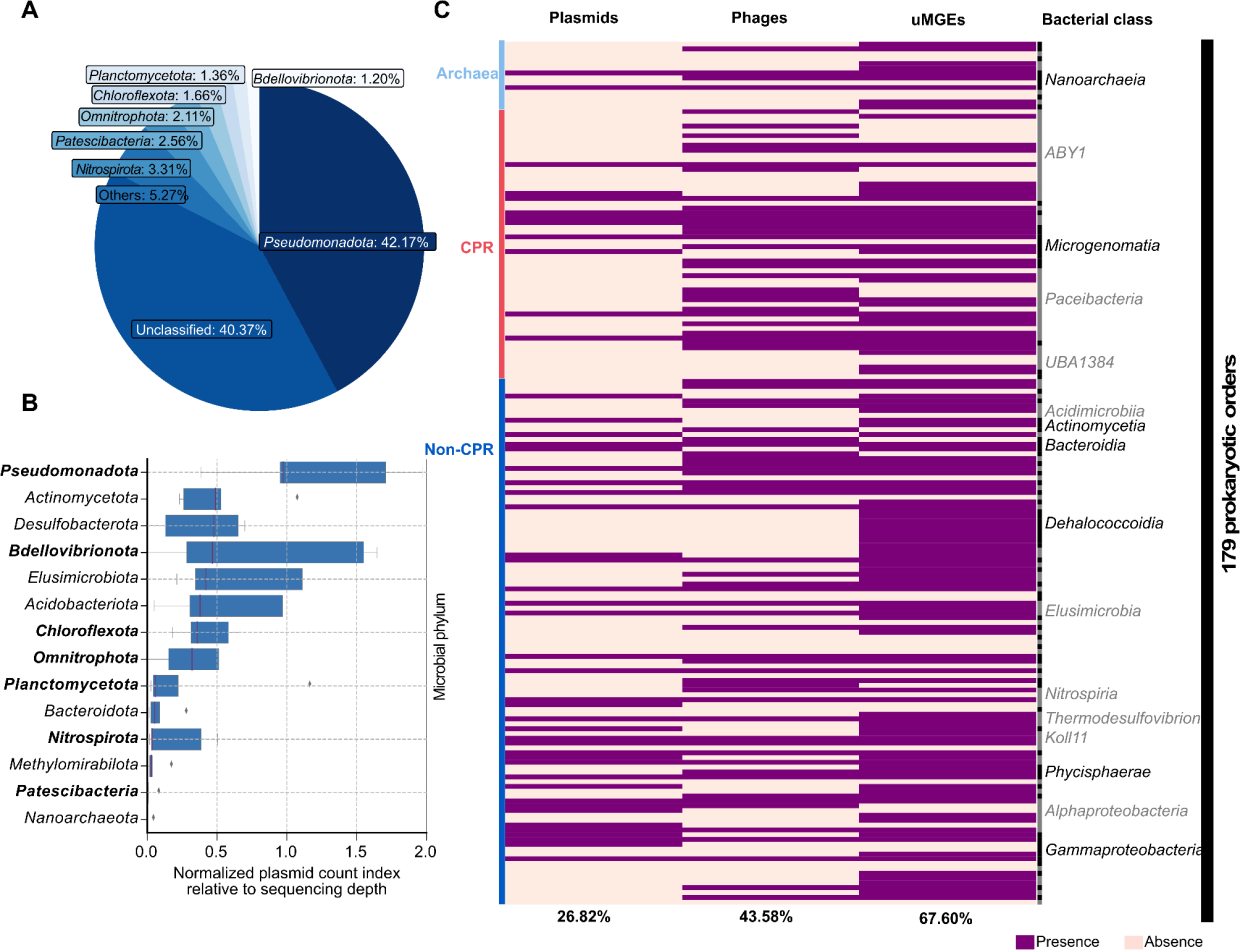
**(A)** Percentage of plasmids associated with microbial hosts at the phylum level. **(B)** The box plots illustrate the distribution of plasmid counts across microbial phylum, normalized for sequencing coverage depth. The central line in each box marks the median, while the box itself spans from the first to third quartiles. Outliers were plotted individually, and four of them exceeding a value of 2 were omitted. The phylum highlighted in black in Fig. 3B correspond to those in Fig. 3A. **(C)** MGEs linked to 179 prokaryotic orders present in groundwater. The presence/absence of MGEs is shown. An occurrence was considered if at least one host per taxonomic order was associated with a specific type of MGE. Only taxonomic classes with at least three orders are highlighted. The percentage of prokaryotic orders associated with at least one MGE sequence is indicated below the figure

Among the MGEs with potential host associations (*n* = 1,729), plasmids and phages represented 22.90% (*n* = 396) and 18.16% (*n* = 314), respectively. In contrast, a larger proportion, 58.94% (*n* = 1,019), corresponded with uMGEs. These MGEs with possible host associations were linked to several microbial lineages, including the most abundant CPR bacteria and archaea found in groundwater (Fig. 2; Supplementary Figure S3). Plasmids have been rarely studied in CPR bacteria, representing a significant gap in our understanding of these ultra-small microbes. Here, we observed that a small fraction (2.56%; 17 out of 664) of plasmids was potentially associated with CPR bacteria (*Patescibacteria* phylum), encompassing 11 orders, 10 of which are found in groundwater (Fig. 3A). In contrast, the highest proportion of plasmid sequences (approximately 42.17%; 280 out of 664) were linked to bacterial lineages within the phylum *Pseudomonadota*, the second most abundant group in groundwater, which includes 17 orders, nine of which are also found in this environment. This implied that the *Pseudomonadota* phylum is the main host of plasmids in this groundwater system. MGEs of the phage type were more frequently associated with CPR bacteria (5.94%; 92 out of 1,549) than with *Pseudomonadota* (3.81%; 59 out of 1,549). We then normalized MGE counts across phyla to account for taxon-specific sequencing effort; subsequent analysis revealed that the phylum *Pseudomonadota* was enriched in plasmids compared to other lineages, while *Patescibacteria* and *Nanoarchaeota* showed a depletion (Fig. 3B), which aligned with our initial findings. In contrast to the previous observation based on absolute counts, CPR bacteria were also found to be less enriched in phages and uMGEs relative to *Pseudomonadota* (Supplementary Figure S4A and B). However, it’s important to note that our focus on plasmid identification may have overlooked phage diversity, so this interpretation should be taken with caution.

After classifying non-redundant MAGs into bacterial prokaryotic orders (*n* = 179), we found that 10 out of 56 (17.86%) groundwater CPR orders were associated with plasmids. In contrast, the ratio for non-CPR bacteria was higher, with 36 out of 109 (33.03%) orders showing associations with plasmids (Fig. 3C). Among archaea, only 2 out of 14 (14.29%) orders were associated with plasmids.

Plasmids potentially hosted by CPR bacteria were smaller on average (mean = 6301.9 bp, SD = 6014.0, *n* = 17) than those potentially hosted by *Pseudomonadota* (mean = 16682.5 bp, SD = 35869.9, *n* = 280). However, this difference was not statistically significant (two-sided Wilcoxon test, adjusted for false discovery rate (FDR), *p* > 0.05). The lack of statistical significance, despite the apparent difference in means, is likely due to the high variability in plasmid sizes within the *Pseudomonadota* phylum, as evidenced by the large standard deviation, as well as the substantial difference in sample sizes between the two groups (Supplementary Figure S5).

We also found 23 MGEs-host associations using spacers, 20 of which were with non-CPR hosts. Most MAG spacers (19/23) matched to phage sequences (Supplementary Table S6). Therefore, the use of spacer-to-protospacer alignments was unreliable to identify potential plasmid-host associations. For example, in refined MAGs, CRISPR-Cas systems arrays with evidence level 3 and 4 were present in only 7.4% (191/2,589) of them.

Furthermore, we evaluated whether our MGEs show similarities with plasmids already reported in public databases, using a query coverage greater than or equal to 90% and a minimum identity of 60%. Only a few MGEs showed similarity with known plasmids (4.34%; 200 out of 4,609), with a mean percentage identity of 92.3%. These matching MGEs were distributed across the three MGE types: plasmids (43.50%), phages (12.00%), and uMGEs (44.50%) (Supplementary Table S1). Most matches were identified within the genera *Sphingobium* (31.00%), *Acinetobacter* (15.50%), *Cupriavidus* (7.50%), *Sphingomonas* (6.00%), *Sphingopyxis* (6.00%), and *Novosphingobium* (4.00%). The predominance of matches to *Sphingobium* and *Acinetobacter* corroborated previous findings in groundwater [43]. Notably, plasmid-related sequences had limited representation in this database (13.10%; 87 out of 664), aligning with the scarcity of matches found in other aquatic microbiomes (1.65%) [33].Thus, the vast majority of MGEs, such as plasmids in groundwater, remain largely uncharacterized.

### Plasmids as well as other MGEs sharing genomic similarities are mostly hosted by phylogenetically related MAGs

**Fig. 4:**
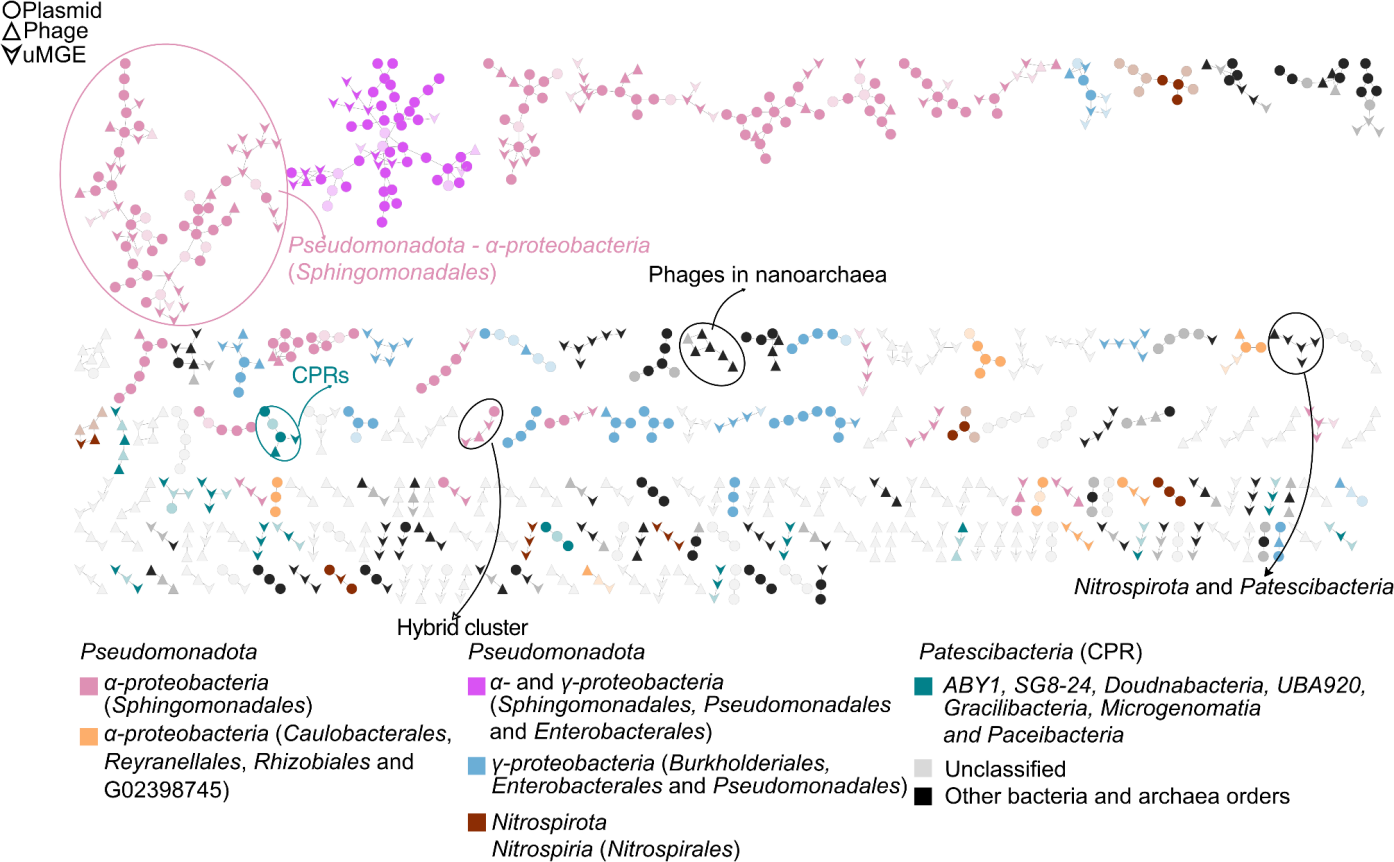
MGE similarity network, with each module containing at least three non-redundant MGEs. The node shape indicates the MGE type. Taxonomic groups at the class/order level are highlighted by different colors in the graph. Nodes with transparent or gray filling lack known host association. Edges connect MGEs contained within one another (≥ 90% sequence identity and ≥ 90% coverage of the smaller plasmid) [54]. Only the most abundant host phyla found in groundwater are shown in the color legend below the figure

MGEs can be organized into modules or clusters of evolutionarily related sequences based on their similarities. To achieve this, we generated a sequence similarity network using the MobMess algorithm, which performed pairwise alignment comparisons of MGE sequences. This approach enabled the grouping of connected sequences into distinct similarity modules. After linking the host to the MGEs (1,729 out of 4,609), we observed that several MGE modules in the similarity network are mostly hosted by phylogenetically closely related bacteria such as *Alphaproteobacteria* (*Sphingomonadales*; 21 out of 190 (11.05%) modules with at least three MGEs) (Fig. 4). Fourteen of those modules were exclusively related to CPR bacteria. These results show a limited host range for closely related MGEs, as has been shown for plasmids [103]. Here, we also showed several examples of MGE modules (4.74%; 9 out of 190) that contained a mix of sequences associated with various taxonomic groups, including *Pseudomonadota* and *Omnitrophota*, as well as CPR/*Patescibacteria* and *Nitrospirota*, which are distantly related at the phylum level (Supplementary Table S5). Taxonomic assignment of OGs (ortholog groups) within the MGEs based on eggNOG, confirmed that closely related MGEs are probably evolutionarily related (e.g., *Sphingomonadales*). In addition, we found that 50.50% of the modules containing at least three non-redundant MGEs were composed of a mix of MGE types and were considered hybrid clusters. While modules composed of plasmid, phage, and uMGE alone, represented 14.21%, 13.16%, and 22.11%, respectively. The presence of hybrid MGE modules underscored the mosaic composition of plasmid, for example (Supplementary Figure S6, Supplementary Data S1). These modules might preserve backbone genes while carrying accessory genes. However, distinguishing core genes from accessory ones can be challenging when MGE sequences are incomplete or have undergone multiple genetic rearrangements. For instance, genes involved in plasmid replication could be considered part of the core genome [104], although those genes are not generally present in all plasmids [11].

### Spatial distribution of microbial communities, plasmids, and other MGEs, and their positive associations

**Fig. 5:**
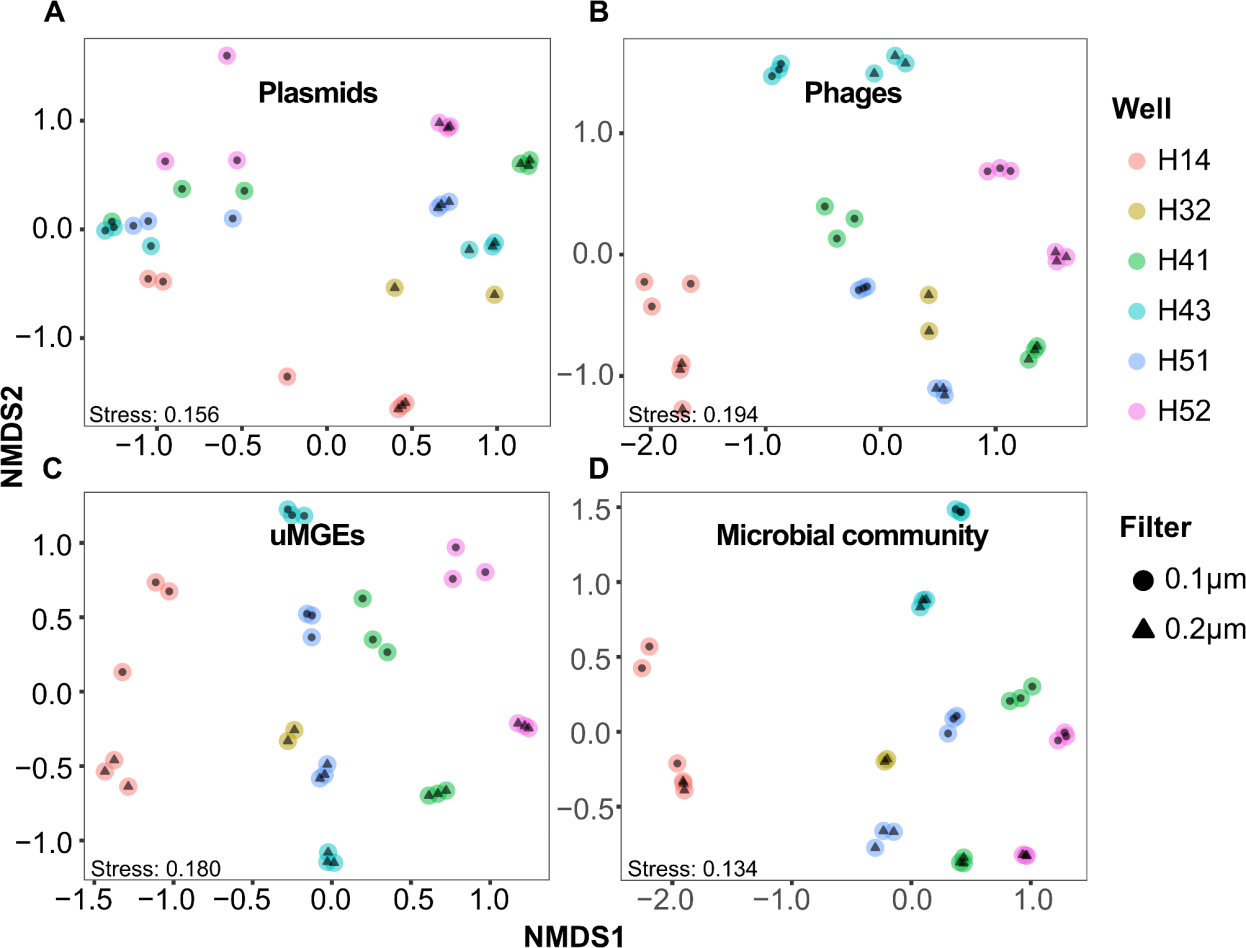
MGEs are shaped by the local groundwater microbiomes. Non-metric multidimensional scaling (NMDS) plots show the local variation in MGEs and microbiome diversity ((**A**), Plasmids, (**B**), Phages, (**C**), uMGEs, and (**D**) Microbial community), based on Bray–Curtis dissimilarity matrices of the normalized coverage of the MGEs and microbial communities across the metagenomic samples. Plots based on Jaccard distances are shown in Supplementary Figure S8. With the sampling site explaining most of the variation (between 47.4% and 77.2%) in the MGEs and microbial community composition (*p* < 0.001)

Given that microbial communities in the Hainich groundwater system exhibit spatial distribution, we wondered whether MGEs, particularly plasmids, show similar trends. To explore this, we assessed the community structures of microbial and MGE types across various sampling sites by analyzing their abundances and estimating dissimilarities. A significant effect of the sampling site was observed on the MGE composition (Bray-Curtis and Jaccard distances; PERMANOVA (*p* ≤ 0.001)). Indeed, plasmids and other MGEs exhibited spatial organization (Fig. 5A, B, **and C**), resembling the spatially distinct microbial communities at the Hainich groundwater sites (Fig. 5D). There was also a significant effect of the filter fractions (0.1 and 0.2 μm). After removing well H32, which contained only two samples from the 0.2 μm fraction, the effects of sampling site and filter remained significant (Supplementary Figure S7). Variations in MGE and microbial community compositions correlated among groundwater wells. We observed local selection of the microbial and the MGE communities in all cases, including non-CPR bacteria and plasmids, for example (Procrustean superimposition (*r* = 0.9393, *p* ≤ 0.001) and Mantel test based on Spearman correlation (*r* = 0.793, *p* ≤ 0.001) (Supplementary Table S7).

Considering the potential co-dependence among bacteria, archaea, and plasmids, we next investigated the interactions between non-redundant MAGs found in groundwater and plasmids by employing association networks. We observed significant positive associations (82.54% between bacteria, archaea, and plasmids) (Supplementary Table S8 and Supplementary Table S9).

Some positive associations between plasmid type and microbes were congruent with the plasmid taxonomic associations identified using nucleotide sequence sharing. For example, five of the 52 positive associations were between plasmids and their previously identified prokaryotic host at the order level in groundwater, suggesting a strong link between these host and plasmids. These orders included *Caulobacterales*, *Burkholderiales*, *Sphingomonadales*,* Brocadiales*, and *Nitrospirales*, several of which are known to be abundant in groundwater [39]. Positive associations were observed between plasmids and CPR/*Patescibacteria*. Interactions were also observed among multiple MAGs and a single plasmid type and vice versa.

While we also explored and observed positive associations for other MGE types, the limitations of our method to predict other MGEs and the potential biases introduced by varying plasmid copy numbers necessitate further exploration to fully understand these relationships. For instance, in the case of phages, 16 out of 131 positive associations were with a known host at the order level, while for uMGEs, this number was 44 out of 199.

### Plasmids are enriched in inorganic ion transport and energy production genes in contrast to other MGEs

**Fig. 6:**
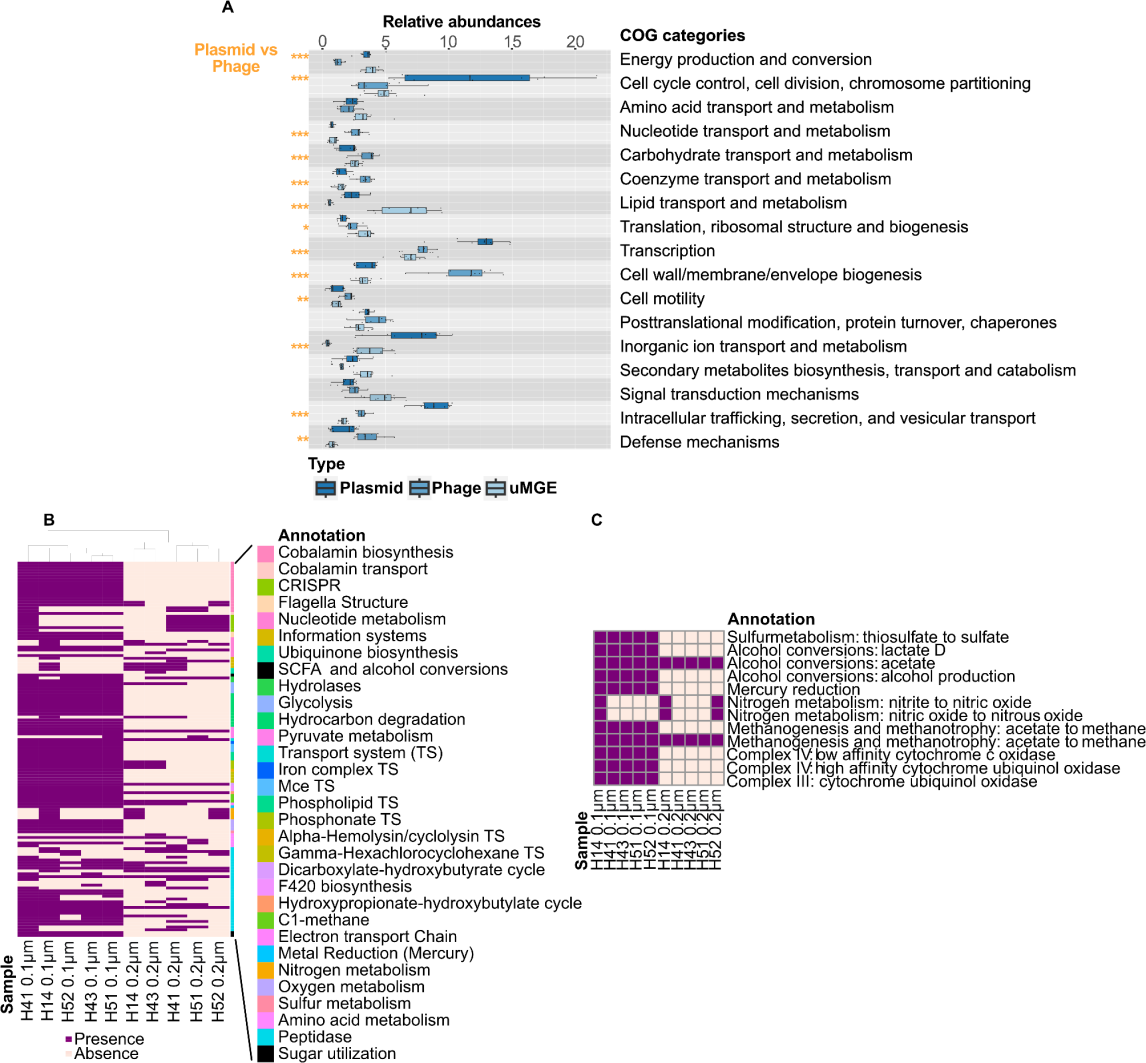
**(A)**. Plasmid functional repertoire compared to other MGEs. Functional enrichment of MGEs based on COG annotations. Differences in functional enrichment between plasmids and phage sequences were tested using a two-sided Wilcoxon test. Statistically significant pairwise comparisons are shown (* *p* < 0.01, ** *p* < 0.001, and *** *p* < 0.0001). **(B)** Metabolic differences across sites and filter fractions in plasmid sequences. The heatmap shows the presence (purple squares) and absence (pink squares) of specific auxiliary metabolic genes (AMGs) in the plasmid sequences across sites. The data corresponding to all the MGEs, phages, and uMGEs are shown in Supplementary Figure S10A, B, and C, respectively. **(C)** Functional annotation of metabolic pathways in plasmid sequences with completeness greater than 0.5

When comparing the enrichment of COG categories among plasmids, phages, and uMGEs, we observed significant enrichment in genes related to inorganic ion transport and energy production in plasmids (Fig. 6A, Supplementary Table S10). Of all the genes in the plasmids, 48% (4,186/ 8,764) have a known function. Functions related to transcription or plasmid own maintenance (mobility) were also enriched in plasmids across all the samples, whereas genes involved in envelope biosynthesis were more common in MGEs identified as phage. Based on additional analysis of auxiliary metabolic genes we observed that plasmids, are functionally different between filter fractions (0.1 and 0.2 μm) (Fig. 6B). Genes related to hydrocarbon degradation, and sulfur metabolism (oxidation of thiosulfate to sulfate) were observed in the plasmids associated with the 0.1 μm filter fraction, while nitrogen metabolism (nitrite to nitric oxic) was present in both fractions. Pathways involved in methanogenesis were observed in plasmids of both filter fractions (acetate to methane) (Fig. 6C). When comparing the plasmid functions potentially hosted by CPR/*Patescibacteria*, we identified a plasmid that carries genes involved in cobalamin biosynthesis, such as *cobS* and *cobT*, which encode cobaltochelatases. Notably, *cobS* was absent in CPR MAGs from our groundwater dataset. This absence in CPR MAGs, alongside the presence of ***cobS*** on a plasmid, suggests that plasmids might compensate for essential metabolic functions. In contrast, plasmids hosted by *Pseudomonadota* not only contained a more complete cobalamin biosynthesis pathway but also encoded genes related to enzymatic functions, including peptidases and key genes involved in methane and hydrocarbon degradation.

We next investigated the relationships between plasmids and environmental variables (pH, dissolved oxygen, ammonium, nitrate, and sulfate) in the Hainich groundwater system, focusing on how these factors influence plasmid composition and gene functions. Using Spearman correlations, we identified 71 out of 664 plasmids (10.7%), which were strongly associated with various environmental parameters (*p* < 0.05; permutations = 999) (Supplementary Figure S9). pH emerged as the dominant driver, with 53 out of 71 (74.65%) plasmids negatively correlated, underscoring its critical role in shaping microbial communities in groundwater systems [99, 105]. The plasmids negatively correlated with pH often contained genes involved in energy production and conversion (COG C), coenzyme transport and metabolism (COG H), inorganic ion transport and metabolism (COG P), and posttranslational modification, protein turnover, chaperones (COG O). In comparison, a plasmid positively correlated with dissolved oxygen encoded a gene for nitric oxide dioxygenase activity (Supplementary Table S11), demonstrating how specific environmental conditions could shape the functional repertoire of plasmids.

**Fig. 7:**
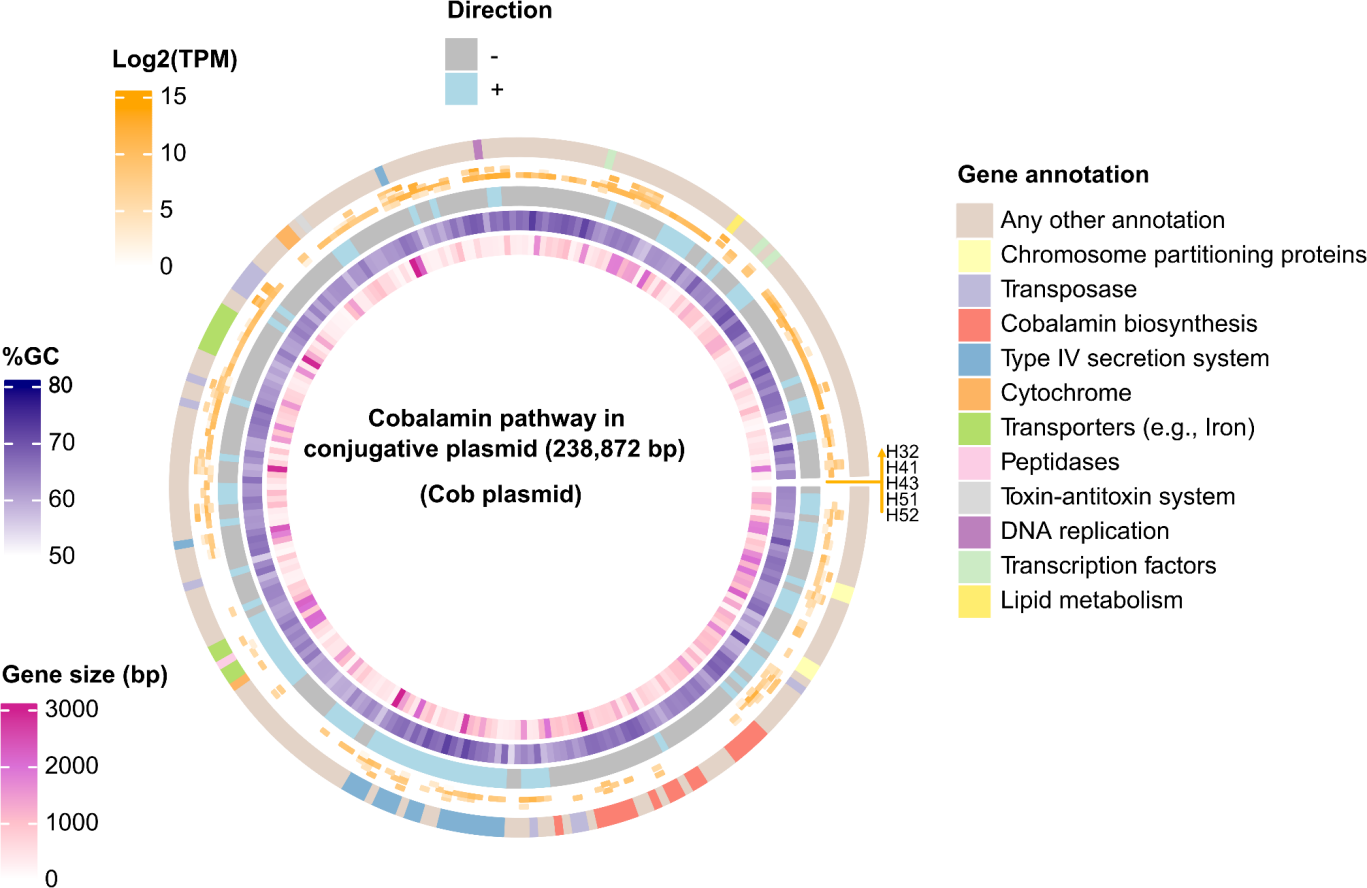
Metatranscriptome data confirmed the transcription of certain genes on the Cobalamin plasmid. From innermost to outermost, the plasmid rings represent gene size, %GC content, gene orientation, the number of transcripts per million across five groundwater wells, and gene functional annotation

A closer examination of the plasmid functions revealed the presence of a conjugative/mobilizable plasmid (238,872 bp) containing genes involved in the aerobic biosynthesis of the corrin ring. This plasmid is probably hosted by members of the *Sphingomonadales*, as shown by the taxonomic annotation of its proteins and the host prediction. Some genes for the anaerobic route of cobalamin were present. Genes involved in the final steps of cobalamin were also present in that plasmid (i.e., nucleotide loop assembly and adenosylation of cobalt). Metatranscriptomic data from a previous sampling time of groundwater obtained from the same wells supported the occurrence of this cobalamin plasmid with transcription of genes involved in the plasmid mobility (secretion systems) and genes related to the corrin ring biosynthesis (*CobG*, *CobH*, *CobM*, and *CobN*) (Fig. 7; Supplementary Table S12). Based on auxiliary metabolic genes, a complete pathway related to the reduction of mercury was also identified in a plasmid (12,176 bp) associated with a *Pseudomonadota* host. This resistance plasmid contains genes that are being transcribed (*merE*, *merD*, *merB*, *merA*, *merP*, and *merR*).

High confidence genes encoding antibiotic resistance mechanisms were absent in plasmids and other MGEs. Only antibiotic resistance genes (ARGs) belonging to the ‘loose’ category were detected in plasmids. These loose hits can represent distantly related homologs to known ARGs or potential false positives, with identity matches below 55% compared to CARD database references. In contrast, genes with at least one strict hit to the CARD database were present in the groundwater MAGs. Strict hits can detect previously unknown variants of known ARGs, indicating the presence of potentially novel antibiotic resistance mechanisms in the groundwater microbial community. No perfect hits (exact matches to curated reference sequences) were found in MAGs. (Supplementary Table S13). This finding emphasizes that plasmids do not play a role in the spread of emerging ARGs in this groundwater system due to their lack of high confidence ARGs.

## Discussion

In this study, we provided an in-depth characterization of the plasmid pool in a pristine, low cell-density groundwater system, predominantly inhabited by ultra-small microbes such as CPR bacteria. In these groundwater systems, plasmids may play a critical role by encoding products that serve as “public goods”, potentially alleviating metabolic constraints of microbial communities and influencing microbial interactions and ecosystem processes. Our analysis revealed a mobilome comprising 4,609 unique sequences. This mobilome consisted of plasmids (14.41%), phages (33.61%), and uncharacterized MGEs (51.98%), many of which remain unidentified with respect to their prokaryotic hosts. Among these, core MGEs (MGEs shared across all sampling sites) represented less than 6% of the total unique sequences. Similarly, core plasmids (plasmids found in all sampling locations) accounted for less than 15% of the total unique plasmid sequences. Remarkably, this groundwater mobilome is 2.85 times larger than that previously reported for low and high heavy metal-contaminated groundwater at the Oak Ridge Reservation [43]. However, plasmids and other MGEs in our pristine system were notably smaller—typically less than 12 kb—compared to those found at Oak Ridge Reservation (mean size 22 kb) [43]. Also, in high-cell density environments, such as human gut, soil and wastewater, plasmids are typically larger, often exceeding twice the median size of those found in groundwater [9, 48, 106, 107].

We also observed a relatively low proportion of plasmid sequences (less than 1%) compared to the total number of metagenomic contigs (mean = 187,464). This finding mirrors results from heavy metal-contaminated groundwater^39^ but contrasts sharply with high-cell density, nutrient-rich environments—such as wastewater treatment plant—that are suspected hotspots for HGT, where plasmid sequences account for approximately 6% of the total metagenome [108]. Moreover, the number of non-redundant plasmid contigs identified in groundwater was significantly lower than in wastewater systems, with 664 detected across 32 metagenomes compared to 10,942 across 78. Differences in plasmid identification methodologies may influence the interpretation of these results [109].

The low abundance of plasmids in groundwater could be attributed to several factors. These include the streamlined genome size and reduced metabolism of host cells compared to eutrophic environments [39], as well as the limited gene flow due to the sparse population of cells (around 1 × 10^5 cells/mL in groundwater [110], compared to the much higher densities in wastewater biofilms [21]). Additionally, the oligotrophic conditions in groundwater, combined with simpler biofilm formation in carbonate rock aquifers [111], hydrological barriers, and a more evenly distributed cell population, likely limit the transfer of larger DNA molecules such as plasmids. In contrast, complex biofilms in nutrient-rich environments—where cells are densely packed and exhibit increased interactions—facilitate more frequent genetic exchange, particularly through conjugation [22]. This higher rate of genetic transfer in biofilms is significantly greater than that observed in dispersed, motile planktonic cells in aquatic environments, where cell-to-cell interactions are more limited [22].

In relation to plasmid lifestyles in groundwater, our analysis revealed a high prevalence of non-mobilizable plasmids (~ 82%). This proportion is higher than those generally reported in other environmental systems, where non-mobilizable plasmids typically represent between 40% and 59% [112] of the total plasmid population, though some other studies report values as high as 90% [42, 106]. The persistence of low cost/non-mobilizable plasmids in groundwater microbes could be favored by the reduced metabolic burden they cause compared to the mobilizable plasmids, which rely on host energy for processes such as conjugation between cells, rather than using natural transformation or transduction [43, 106, 113]. This observation aligns with the conditions contributing to the low abundance of plasmids in groundwater, emphasizing how ecological constraints limiting plasmid presence could also shape their mobility within groundwater microbiomes.

Building on these insights into plasmid lifestyles, we further investigated their host associations and distribution across groundwater microbiomes. Nearly 59.64% of plasmids were successfully linked to their host, representing a higher proportion of plasmid-host connections compared to those observed in the human gut microbiome, where these associations typically range between 21% and 36.03% [29, 114]. This increased proportion of plasmid-host linkage likely reflects the use of extended databases in our analysis, which incorporated data from diverse environments, thereby enhancing the accuracy of plasmid-host association predictions. Here, plasmids were predominantly hosted by bacteria with larger genomes, particularly within the phylum *Pseudomonadota* (42.17%) and the class *Alphaproteobacteria* (27.11%). *Alphaproteobacteria* emerged as the primary hosts of plasmids in low-polluted groundwater, contrasting with *Beta/Gammaproteobacteria*, which were the most frequent hosts in highly polluted groundwater [43] and wastewater [48]. Other host phyla identified included *Nitrospirota* (3.31%), CPR bacteria/*Patescibacteria* (2.56%), and *Omnitrophota* (2.11%). Notably, this order does not reflect the relative abundance of these taxa in the Hainich groundwater system, which is dominated by CPR/*Patescibacteria* (23–79% of the community), with other phyla present only secondarily [39]. Our findings provide important insights into the underexplored role of plasmids in CPR bacteria. Although plasmids were identified in a small proportion of CPR bacteria, the scarcity could be attributed to their symbiotic lifestyles, reduced genome sizes, and simplified metabolic capabilities [115]. These traits may render CPR bacteria less prone to HGT compared to taxa with larger genomes, which are generally more involved in HGT and can contain several and larger plasmids [116].

Despite their low representation among plasmid hosts, CPR bacteria may still benefit from plasmids and other MGEs. The mechanisms by which abundant taxa such as CPR bacteria interact with plasmids and harness their functions, particularly in nutrient-limited groundwater environments, remain an open and intriguing question. Understanding how these ultra-small microbes integrate MGEs into their lifestyle could shed light on the ecological roles of plasmids in shaping microbial interactions and survival strategies in low-biomass systems. However, studying these associations is challenging due to the transient and random linkages between plasmids and their microbial hosts, each following distinct evolutionary trajectories. This complexity likely contributes to the absence of clear genomic signatures linking plasmids to their hosts when using tools like Mash and iPHoP on microbial databases. Additionally, the rarity of CRISPR-Cas systems in groundwater microbe, particularly in CPR bacteria [117], further complicates efforts to identify host associations using spacer-to-protospacer alignments. Improving metagenomic sequencing with long-read and Hi-C, along with spatial mapping of MGEs and hosts using fluorescence in situ hybridization (FISH), could help uncover more of these interactions [47].

Our results of plasmid distribution across phylogenetic groups provide further insight into how plasmids and other MGEs are mobilized within groundwater microbiomes. Specifically, we found that plasmids within the same similarity modules were more likely to be hosted and mobilized by bacteria within the same family (e.g., *Alphaproteobacteria/Sphingomonadaceae*, 11.05%). In contrast, modules composed of sequences linked to different hosts at the phylum level were relatively scarce, representing approximately 4.74% of the modules composed by at least three MGEs with a median size of 7.5 kb. Still, the persistence of broad-range similarity modules in groundwater remains uncertain. In contrast, broader range plasmids recognized to span across different phyla in human microbiomes (1.5%), were generally larger (median = 30 kb) and often carried ARGs [118]. The rarity of these broad-range plasmids, coupled with the prevalence of similar plasmids shared among closely related microbes, suggested the presence of genetic barriers within microbial populations [11, 29]—a topic that remains to be explored in groundwater environments.

Similarity modules, defined as those containing at least three non-redundant MGEs, predominantly showed a hybrid composition (50.50%) consisting of a mix of plasmids, phages, and uMGEs. This highlights the mosaic structure of MGEs [119, 120], including plasmids, which can be composed of genetic components from different sources that change in function of the host ecology. In addition, when we accounted for all the identified modules, including those with fewer than three MGEs per module, the majority were composed of one single non-redundant MGE, (3,188 out of 3,643, or 87.51%), which further reinforced the uniqueness of our groundwater mobilome.

The diverse composition and uniqueness of groundwater mobilome could suggest that plasmids and other MGEs, like microbial communities, appear to undergo local selection under similar ecological constraints, with the sampling site explaining most of the observed variation (between 47.4 and 77.2%). Previous research on the Hainich groundwater system highlight the importance of the hydrochemistry (35.0%) and the spatial distances (18.6%) in shaping microbial community composition of each individual well [99]. Similarly, in other environments, plasmid composition and resistomes have been shown to vary due to local selection pressures influenced by microbiome diversity and abiotic conditions [121, 122]. Incorporating additional environmental factors, such as seasonality and microbial dispersal, could shed light on plasmid stability and dynamics in pristine groundwater systems. Understanding these dynamics is particularly important for evaluating whether the groundwater mobilome is predominantly shaped by selective forces over neutral processes, a question that remains open to further investigations [106].

To further delve into these ecological associations, network analysis between plasmid and bacteria indicated that plasmids for example showed strong positive associations with taxonomic group that commonly co-occurred and potentially interacted in groundwater, such as *Nitrospirales*, CPR/*Paceibacteria*, *Pseudomonadota*, and *Nanoarchaea* [39]. While co-occurrence network analyses have generally been used to study MGE-host ecological associations, particularly in the context of phages [123], their application to plasmids and in groundwater remained limited. Network analysis, complemented with functional analyses, could further aid in unveiling the ecological implication of these dominant positive associations. For instance, plasmid-host interactions in wastewater revealed that beneficial plasmids are more likely to interact with a higher number of hosts than non-beneficial plasmids [48].

In addition to these ecological patterns, we explored the functional roles of plasmids as carriers of key ecological functions. Our analysis indicated that plasmids, including those with positive associations, showed enrichment in genes involved in inorganic ion transport, energy production, and conversions, contrasting with other MGEs. This pattern aligned with observations from marine and freshwater plasmids [112]. Regarding the link between plasmids and environmental variables, pH had the strongest effect on plasmids (affecting 10.69% of them), reinforcing the role of pH in shaping microbial communities, as previously observed, and extending this influence on plasmids as well. A key finding of our study is the occurrence of a conjugative plasmid involved in cobalamin ring biosynthesis across multiple groundwater sites. This plasmid was not linked to a known-host and was identified in a community predominantly composed of microbes passing the < 0.2 μm filter. The expression of several genes localized on this plasmid, as indicated by prior metatranscriptome data [44], suggested its potential metabolic activity. Cobalamin is essential as a cofactor in various microbial cellular processes, including environmentally significant cycles such as carbon fixation and acetogenesis via the Wood-Ljungdahl (WL) pathway [124–126]. Notably, the abundant *Nitrospirota*, thought to fix CO_2_ in groundwater via WL [36], exhibited positive and strong associations with other plasmids. Plasmid-derived products could serve as key public goods, facilitating cross-feeding interactions within communities enriched in auxotrophic and small microbes, thereby easing the metabolic limitations. Additionally, genes located in some plasmids could complement metabolic pathways on the host chromosome. For instance, plasmids containing cobalamin-related genes were found in symbiotic microbes harboring complementary genes in their chromosome [127].

Moreover, we identified a complete pathway for mercury (Hg) reduction in a plasmid associated with a *Pseudomonadota* host. Hg is a highly volatile and globally distributed element, with sedimentary rocks serving as natural reservoirs that could potentially expose groundwater microbial communities to this element [128]. However, no elevated Hg concentrations are known in the Hainich CZE groundwater or host rock, which is predominantly composed of carbonate lithologies typically low in heavy metal content with Hg of two orders of magnitude below the ppm range [129]. The presence of Hg resistance genes likely reflects legacy genetic elements or co-selection under other environmental stressors [130], rather than a direct response to local mercury contamination. This plasmid could facilitate the mobilization of Hg resistance genes via HGT, impacting both pristine and low-impacted groundwater systems [43]. In addition to Hg-related functions, plasmids carried metabolic genes involved in methanogenesis - a known function in groundwater microbes [131]. While these specific functions underscore the importance of plasmids in microbial metabolism, a substantial proportion of plasmid genes (52%) remained unannotated. This aligns with findings from other environments, where unannotated genes account for over 60% of plasmid sequences [27, 121]. The combination of well-characterized and unannotated genes emphasizes the dual role of plasmids: as key players in microbial ecosystem functionality and as reservoirs of genetic potential yet to be understood.

Lastly our groundwater plasmids lacked emerging anthropogenic-related ARGs. Plasmids are known for being carriers and spreaders of ARGs and there is evidence of ARGs’ global distribution [132]. For instance, rapid dispersion of non-native plasmids, even across taxonomic boundaries, was reported from groundwater-fed sand filter microbiomes suggesting that groundwater microbes may serve as a vector for the propagation of ARGs [133]. The absence of any ARGs on plasmids and other MGEs in pristine groundwater contrasted with observations in MGEs recovered from polluted groundwater [42, 43], and nutrient-rich environments, in which MGEs harboring ARGs are strongly associated with thriving copiotrophic bacteria [134–136]. This finding highlights the need to preserve the pristine nature of these ecosystems and is aligned with the scarcity of ARGs in low human activity environments [137, 138].

While this study primarily focused on plasmids, it became evident that distinguishing these genetic elements from others, such as phage sequences and uMGEs, posed a significant challenge. The reliance on short genomic reads and the inability of existing plasmid prediction tools to exclusively identify plasmid sequences were notable limitations. Plasmids often carry insertion or repeat sequences, complicating their assembly and recovery based on short sequence reads [102]. Therefore, the diversity and completeness of the plasmids in groundwater might still be underestimated based on the current dataset. Upon subsequent reclassification, distinct MGE types emerged, each displaying marked differences in gene functionality. Given current bioinformatics bottlenecks, it may be more effective to explore the mobilome as a whole [43, 67], acknowledging the distinct biological features and ecological roles of different MGE types. Despite applying multiple host prediction strategies, the inherent constraints of short-read sequencing and existing MGE binning methods likely limited the resolution of true host associations, which may have impacted the accuracy of our findings. Although the extent of these missing associations remains uncertain in this study, previous work suggests that fewer than 29% of plasmids are successfully binned from short-read metagenomic datasets [102]. Integrating improved DNA extraction protocols for MGEs with long-read sequencing and proximity-ligation methods such as Hi-C (High-throughput chromosomal conformation capture) holds promise for expanding the detectable taxonomic breadth of MGEs and enhancing the resolution of host–element associations, including those that span across different phyla [30, 139].

## Conclusions

Our study opens a path to the unexplored mobilome in groundwater ecosystems and their interactions with co-occurring prokaryotic hosts, including the abundant *Pseudomonadota*,* Nitrospirota*, and CPR*/Patescibacteria*. It sheds light on the role of plasmids in facilitating gene spread in low cell density and oligotrophic environments among phylogenetically related microbes, as well as their potential ecological role as carriers of public goods including a cofactor. Further research is essential to deepen our understanding of these interactions and their ecological and evolutionary implications.

## Electronic supplementary material

Below is the link to the electronic supplementary material.

Supplementary Material 1

Supplementary Material 2

Supplementary Material 3

Supplementary Material 4

## Data Availability

The raw metagenomic data for the 32 metagenomes and the individual sample assemblies are deposited on the European Nucleotide Archive (ENA) under the projects PRJEB36505 and PRJEB36523, respectively. The Metagenome-assembled genomes (MAGs) used in this study are deposited on the NCBI under the project PRJEB36505. MGEs sequences and unrefined MAGs are publicly available in Zenodo (https://doi.org/10.5281/zenodo.14500014).
