# Supplementary material for: Unveiling plasmid diversity and functionality in pristine groundwater
Source: Environ Microbiome. 2025 Apr 24;20:42. doi: 10.1186/s40793-025-00703-8 (PMC12023590; doi:10.1186/s40793-025-00703-8)
Supplement: Supplementary file 1 — Supplementary Material 1 [file 40793_2025_703_MOESM1_ESM.pdf]

Captions for supplementary tables

Supplementary Table 1. Non-redundant MGE sequence information. This table details the sequence features of 4,609 non-redundant MGEs, categorized as plasmids, phages, and uMGEs. The methods used for MGE classification are included, along with data on the conservation of MGEs within filter fractions and across different sampling sites. Additionally, plasmids containing genes involved in mobilization are listed.

Supplementary Table 2. MAGs used as references. Table 2A contains the genomic characteristics of the non-redundant and refined MAG genome sequences, whereas Table 2B contains the non-refined genomes prior to dereplication, excluding the selected refined MAGs.

Supplementary Table 3. Scaling factors for normalization of MAG and MGE coverages in the metagenomic and metatranscriptomic samples.

Supplementary Table 4. Normalized read coverage of MGEs in the metagenomic samples. Tables 4A, 4B, and 4C correspond to plasmid, phage, and uMGE coverage, respectively.

Supplementary Table 5. MGE-host assignment based on shared sequences using Mash and IPHoP. The table indicates the MGEs that match to the microbial host.

Supplementary Table 6. MGE-host assignment using spacer-to-protospacer alignments (6A) and spacer sequences associated with a microbial host (6B).

Supplementary Table 7. Procrustes and Mantel results. The table contains the correlation values and significance between MGEs (plasmids, phages, and uMGE) and microbes (all microbes, non-CPR, CPR, and archaea).

Supplementary Table 8. Association counts between microbes and MGEs. The values in the positive or negative columns correspond to associations greater than 0.1 and less than -0.1, respectively. The median and Interquartile range (IQR) values were estimated based on the association values. A positive value indicates co-dependence between individuals (see **Supplementary Table S9**).

Supplementary Table 9. MGEs-host association values. Tables 9A, 9B, and 9C show the association values for plasmids, phages, and uMGE, respectively.

Supplementary Table 10. COG annotation for each MGE class. The table shows the relative abundance of each COG category in the MGEs per sample.

Supplementary Table 11. Plasmids, phages, and uMGEs with significant correlations to environmental variables (11A). Functional annotations of genes from correlated MGEs (11B).

Supplementary Table 12. Transcription data (tpm) of genes in MGEs (Table 12A). Gene annotations and transcription data for plasmids involved in cobalamin and mercury (Hg) metabolism. Table 12B shows the cobalamin plasmid, and Tables 12C and 12D show the Hg plasmids. The TPM counts are provided for each metatranscriptome sample.

Supplementary Table 13. ARG identification in MGEs and MAGs. Tables 13A, 13B, 13C, and 13D contain the hits in the ARG databases for plasmids, phages, uMGEs, and MAGs genes. All hits identified for MGE genes using RGI and the CARD databases were assigned to the loose category.

Captions for supplementary figures

Supplementary Figure S1. Bioinformatic workflow used in MGE identification.

Supplementary Figure S2. Density plots showing the distribution of MGE length (in base pairs, bp) and GC content. MGEs longer than 50,000 bp were excluded to improve readability.

Supplementary Figure S3. MGEs are distributed across several archaea orders. The unrooted phylogenetic tree was built based on a protein-concatenated alignment of refined MAGs generated using the tool GTDB-Tk.

Supplementary Figure S4. The box plots illustrate the distribution of phage (A) and uMGE (B) counts across microbial phyla, normalized for sequencing coverage depth. The central line in each box marks the median, while the box itself spans from the first to third quartiles. Outliers were plotted individually. Those exceeding a value of 2 for phage and 8 for uMGE were omitted from the plot. We estimated the p-values between taxonomic groups using two-sided Wilcoxon test and correcting by FDR, but the results were not significant after correction.

Supplementary Figure S5. Distribution of plasmid sizes across major host-associated bacterial phyla**.**

Supplementary Figure S6. Syntenic block of genes shared between MGEs within a selected similarity module. The alignment and plot of the selected MGEs were performed in Mauve with default settings[140]. CDS annotations for each MGE are described in **Supplementary Data S1**.

Supplementary Figure S7. NMDS plots show the local variation in MGEs and microbiome diversity **(A)** Plasmids, **(B)** Phages, **(C)** uMGEs, and **(D**) Microbial community, based on Bray–Curtis dissimilarity matrices of the normalized coverage of the MGEs and microbial communities across the metagenomic samples, with the 0.2 μm filter fraction from well H32 excluded.

Supplementary Figure S8. NMDS plots show the local variation in MGEs and microbiome diversity ((**A**), Plasmids, (**B**), Phages, (**C**), uMGE, and (**D**) Microbial community), based on Jaccard distance of the normalized coverage of the MGEs and microbial communities across the metagenomic samples.

Supplementary Figure S9. Heatmaps showing Spearman correlations between MGEs (plasmids (A), phages (B), and uMGEs (C)) and environmental variables.

Supplementary Figure S10. Metabolic differences across sites and filter fractions in all the MGE (A), phage (B), and uMGE (C) sequences. The heatmap shows the presence (purple squares) and absence (pink squares) of specific auxiliary metabolic genes (AMGs) in the MGE sequences across sites.
