# Supplementary material for: Unveiling plasmid diversity and functionality in pristine groundwater
Source: Environ Microbiome. 2025 Apr 24;20:42. doi: 10.1186/s40793-025-00703-8 (PMC12023590; doi:10.1186/s40793-025-00703-8)
Supplement: Supplementary file 2 — Supplementary Material 2 [file 40793_2025_703_MOESM2_ESM.pdf]

Supplementary Figures

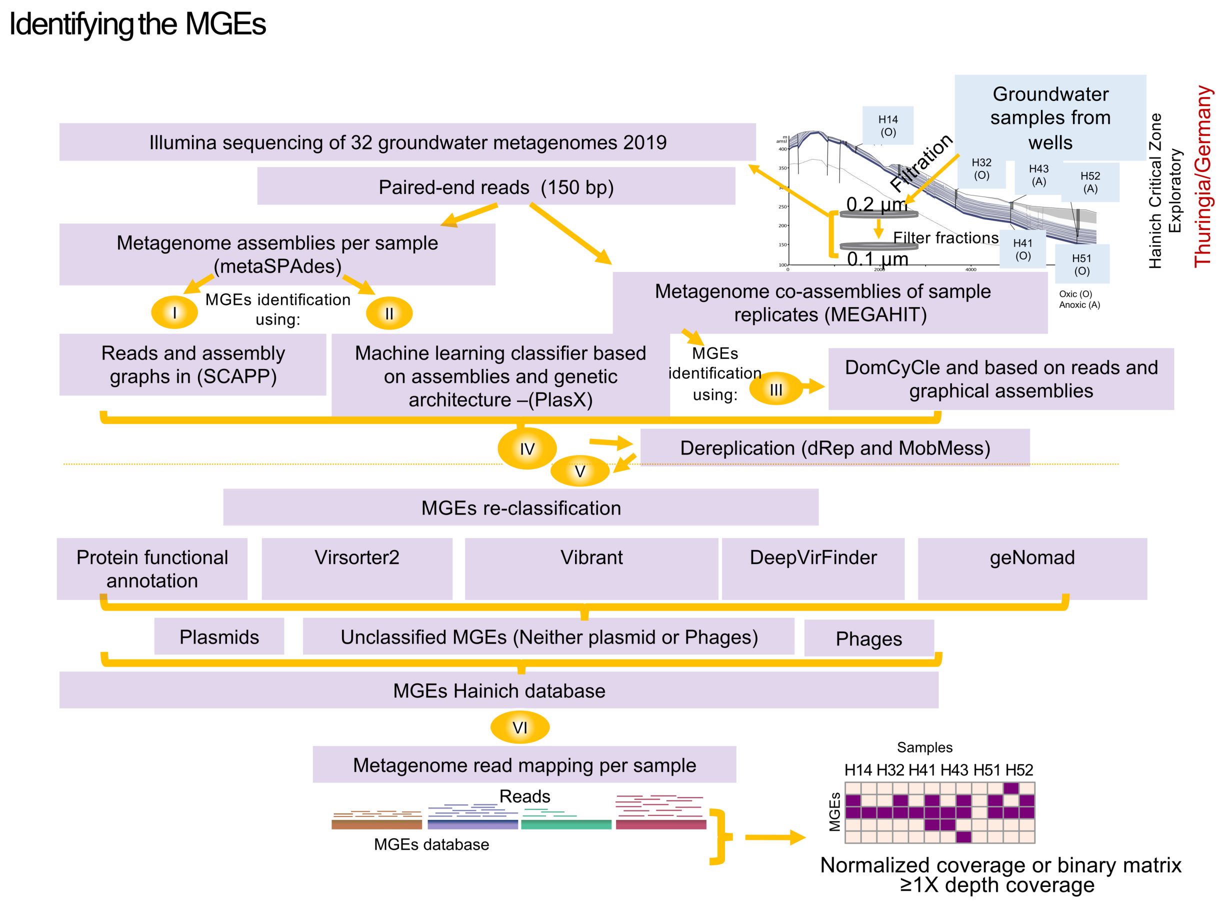

Supplementary Figure S1. Bioinformatic workflow used in MGE identification.

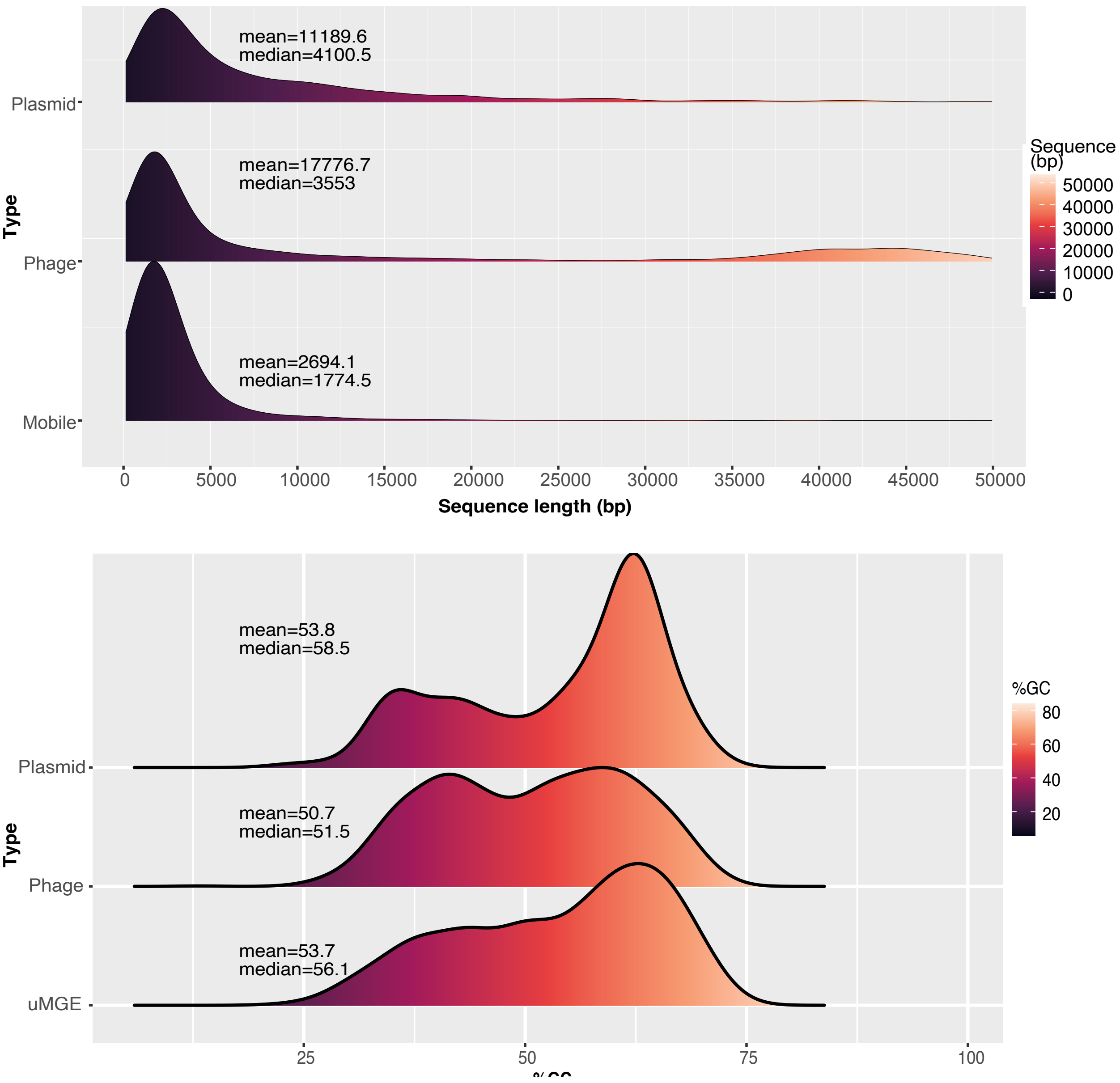

Supplementary Figure S2. Density plots showing the distribution of MGE length (in base pairs, bp) and GC content.

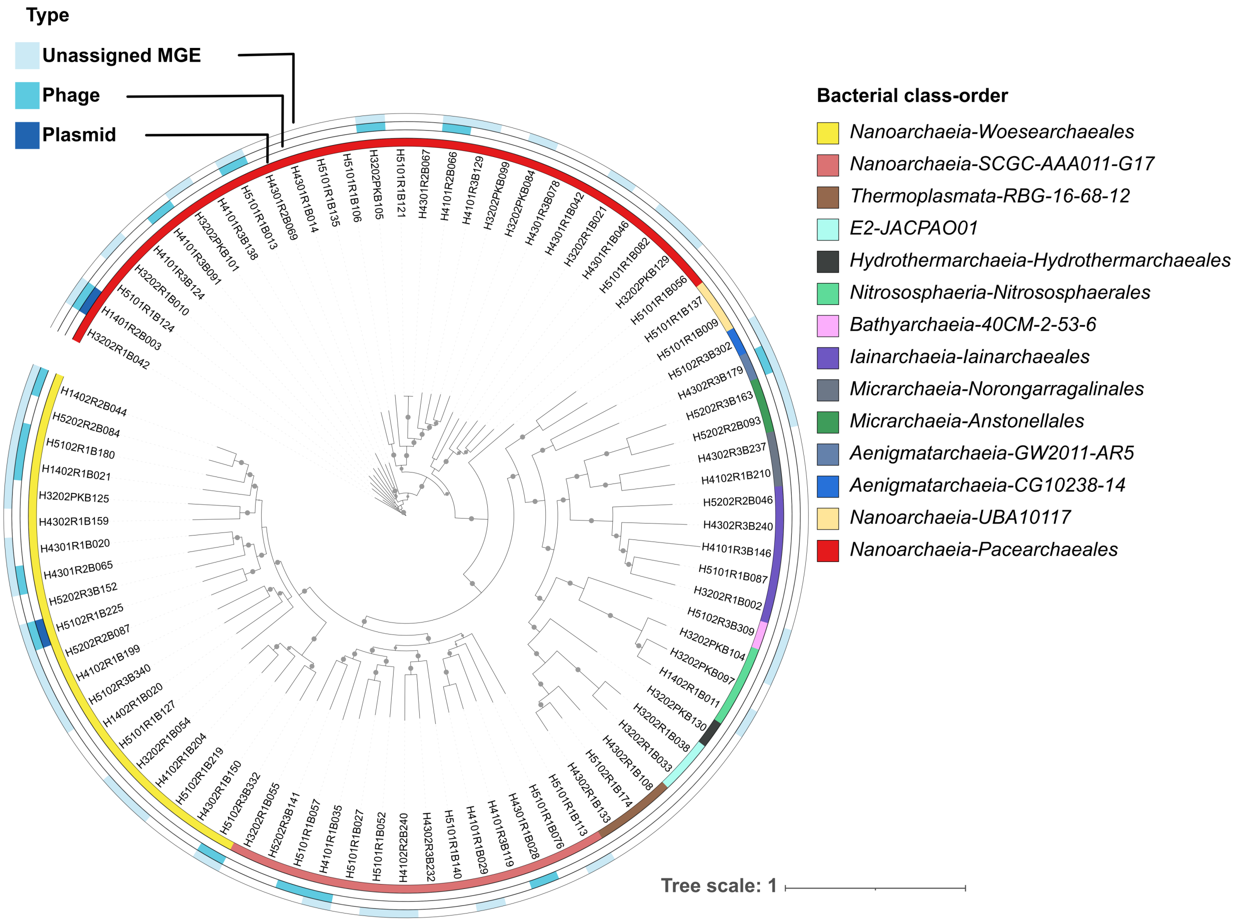

Supplementary Figure S3. MGEs are distributed across several archaea orders. The unrooted phylogenetic tree was built based on a protein-concatenated alignment of refined MAGs generated using the tool GTDB-Tk.

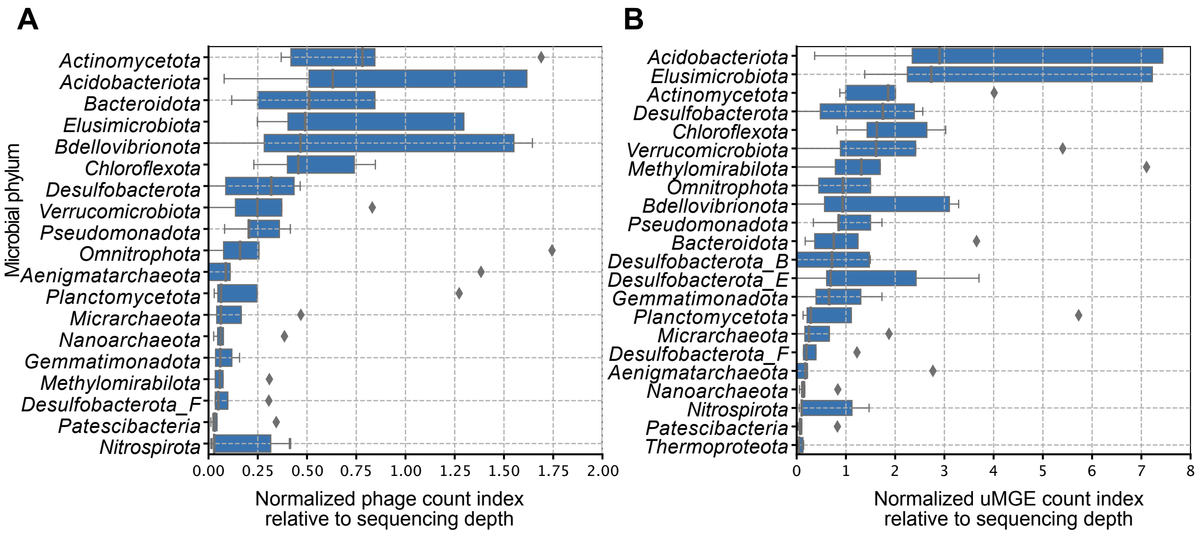

Supplementary Figure S4. The box plots illustrate the distribution of phage (A) and uMGE (B) counts across microbial phylum, normalized for sequencing coverage depth. The central line in each box marks the median, while the box itself spans from the first to third quartiles. Outliers were plotted individually. Those exceeding a value of 2 for phage and 8 for uMGE were omitted from the plot.

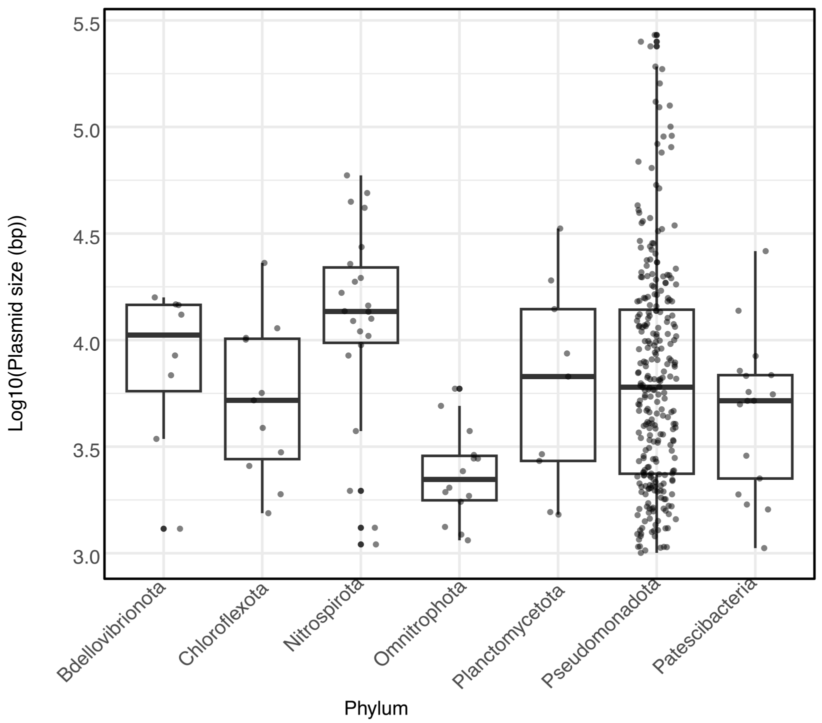

Supplementary Figure S5. Distribution of plasmid sizes across major host-associated bacterial phyla**.**

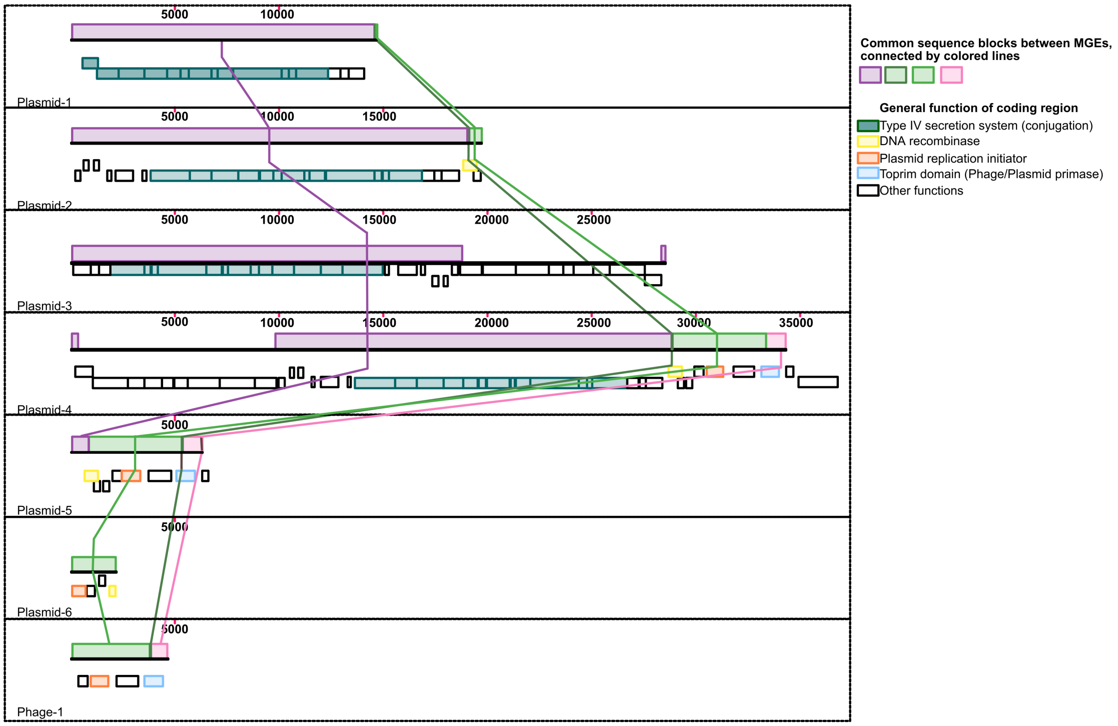

Supplementary Figure S6. Syntenic block of genes shared between MGEs within a selected similarity module. The alignment and plot of the selected MGEs were performed in Mauve with default settings [140]. CDS annotations for each MGE are described in **Supplementary Data S1**.

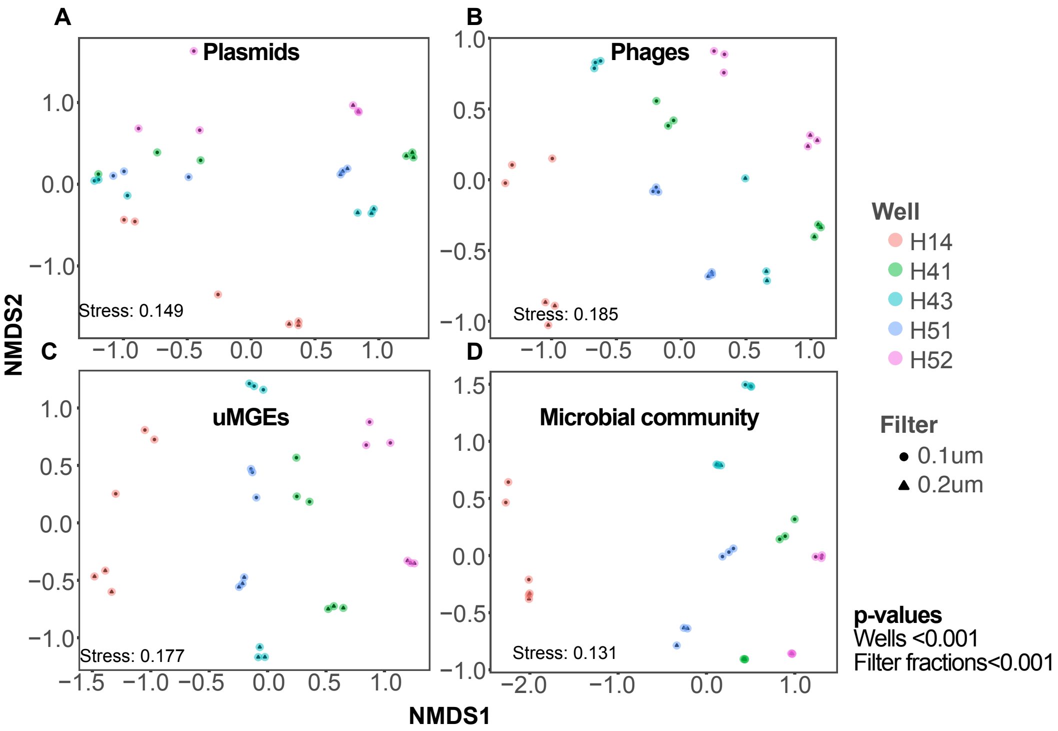

Supplementary Figure S7. NMDS plots show the local variation in MGEs and microbiome diversity ((**A**), Plasmids, (**B**), Phages, (**C**), uMGE, and (**D**) Microbial community), based on Bray–Curtis dissimilarity matrices of the normalized coverage of the MGEs and microbial communities across the metagenomic samples, with the 0.2 µm filter fraction of well H32 excluded.

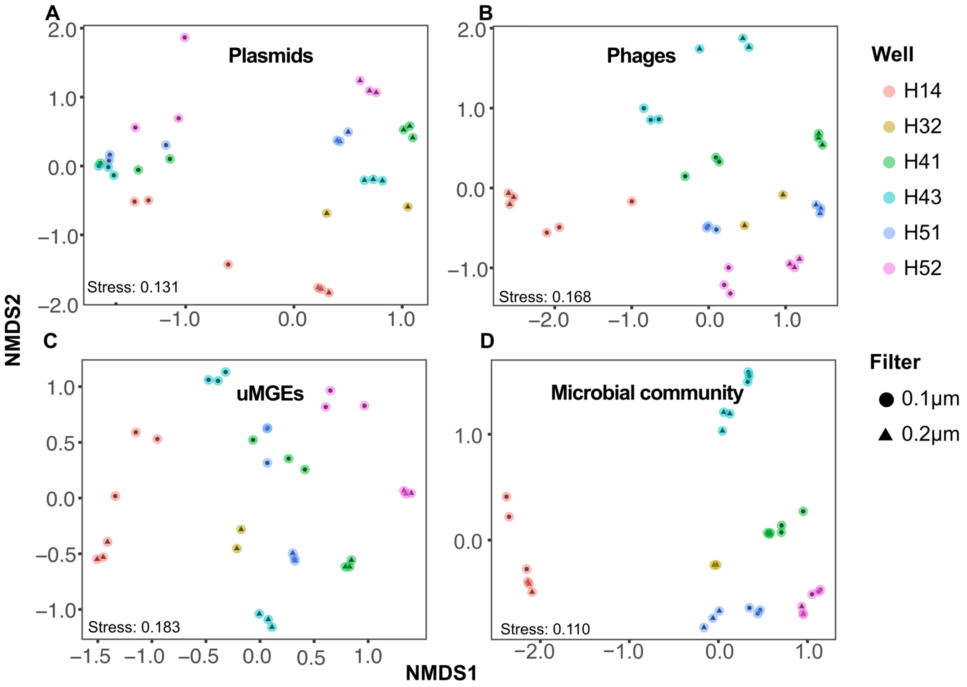

Supplementary Figure S8. NMDS plots show the local variation in MGEs and microbiome diversity ((**A**), Plasmids, (**B**), Phages, (**C**), uMGE, and (**D**) Microbial community), based on Jaccard distance of the normalized coverage of the MGEs and microbial communities across the metagenomic samples.

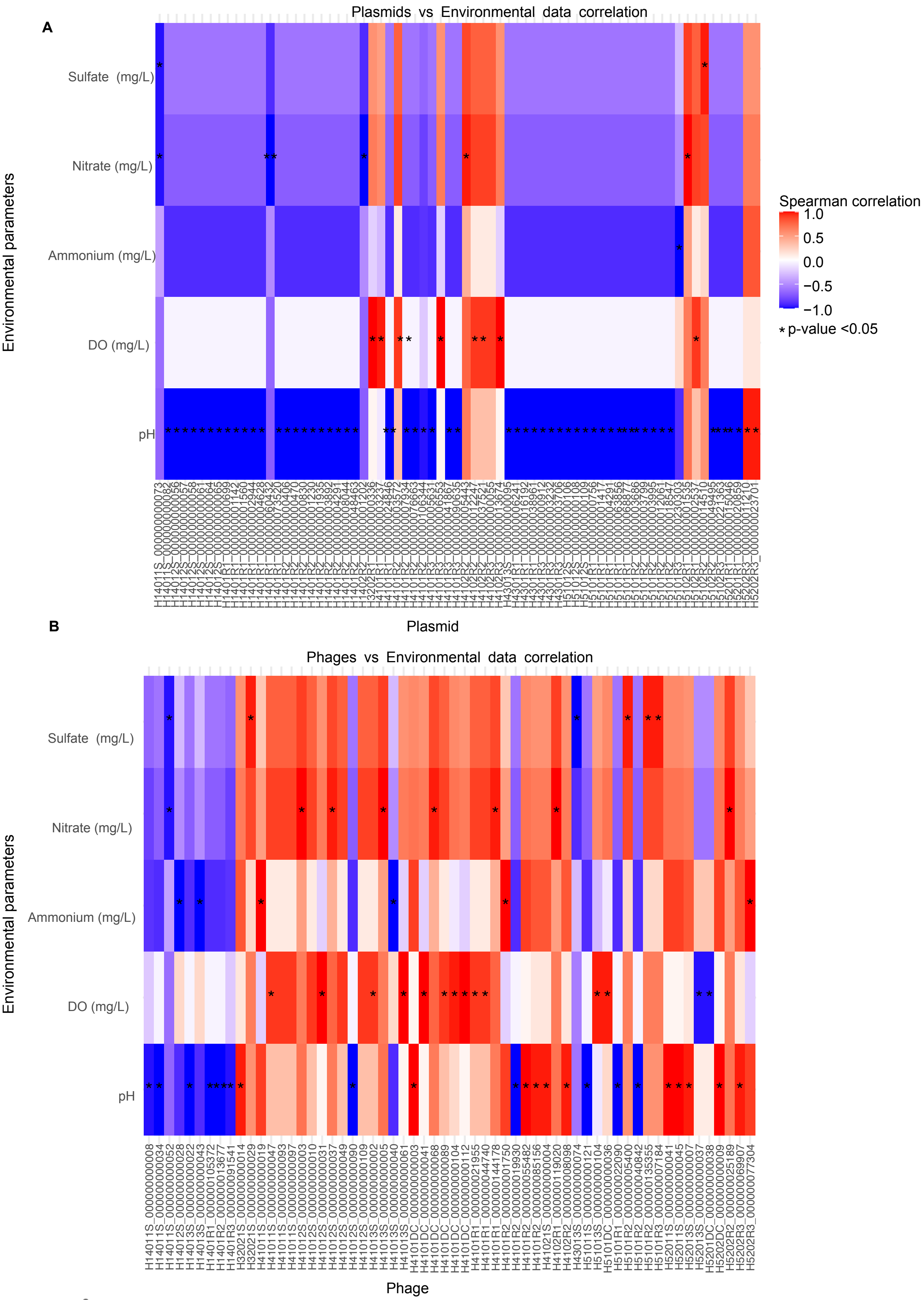
A
Plasmids vs Environmental data correlation
Sulfate (mg/L)
Nitrate (mg/L)
Ammonium (mg/L)
DO (mg/L)
pH
Environmental parameters
Plasmid
Spearman correlation
1.0
0.5
0.0
−0.5
−1.0
* p-value <0.05
B
Phages vs Environmental data correlation
Sulfate (mg/L)
Nitrate (mg/L)
Ammonium (mg/L)
DO (mg/L)
pH
Environmental parameters
Phage

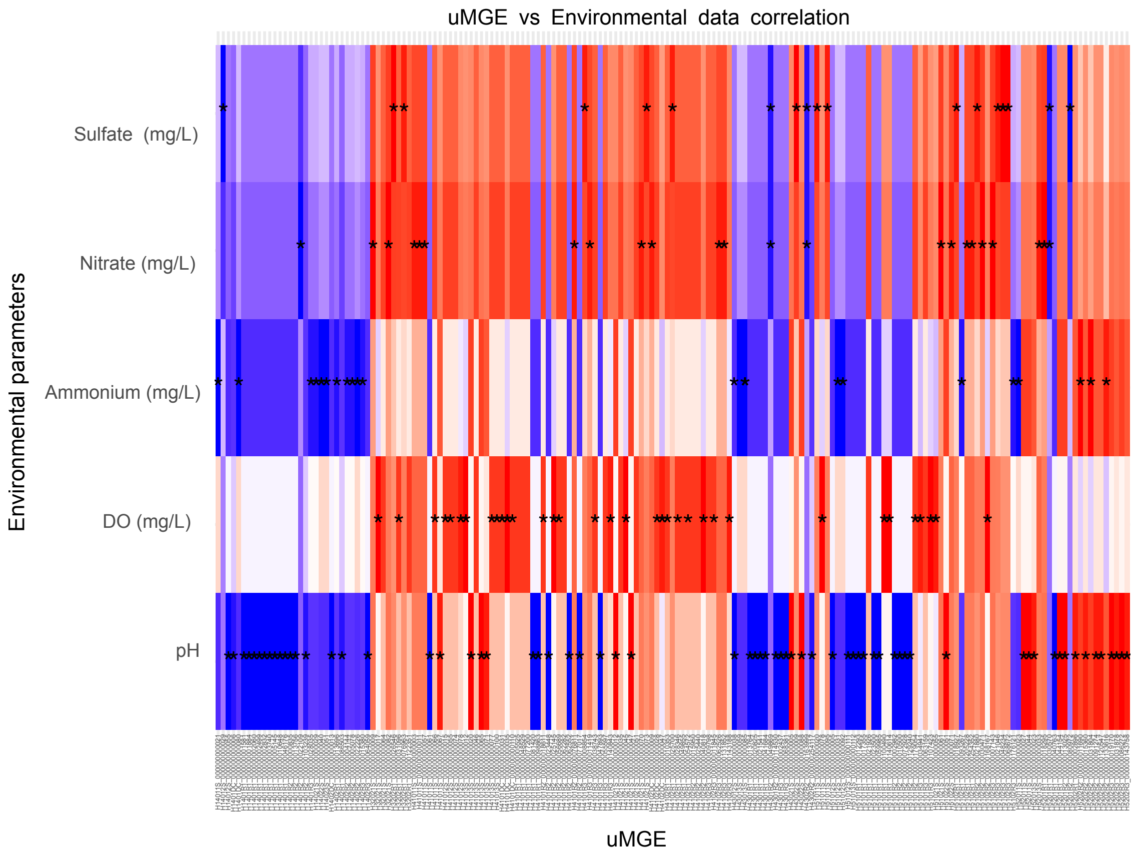

Supplementary Figure S9. Heatmaps showing Spearman correlations between MGEs (plasmids (A), phages (B), and uMGEs (C)) and environmental variables.

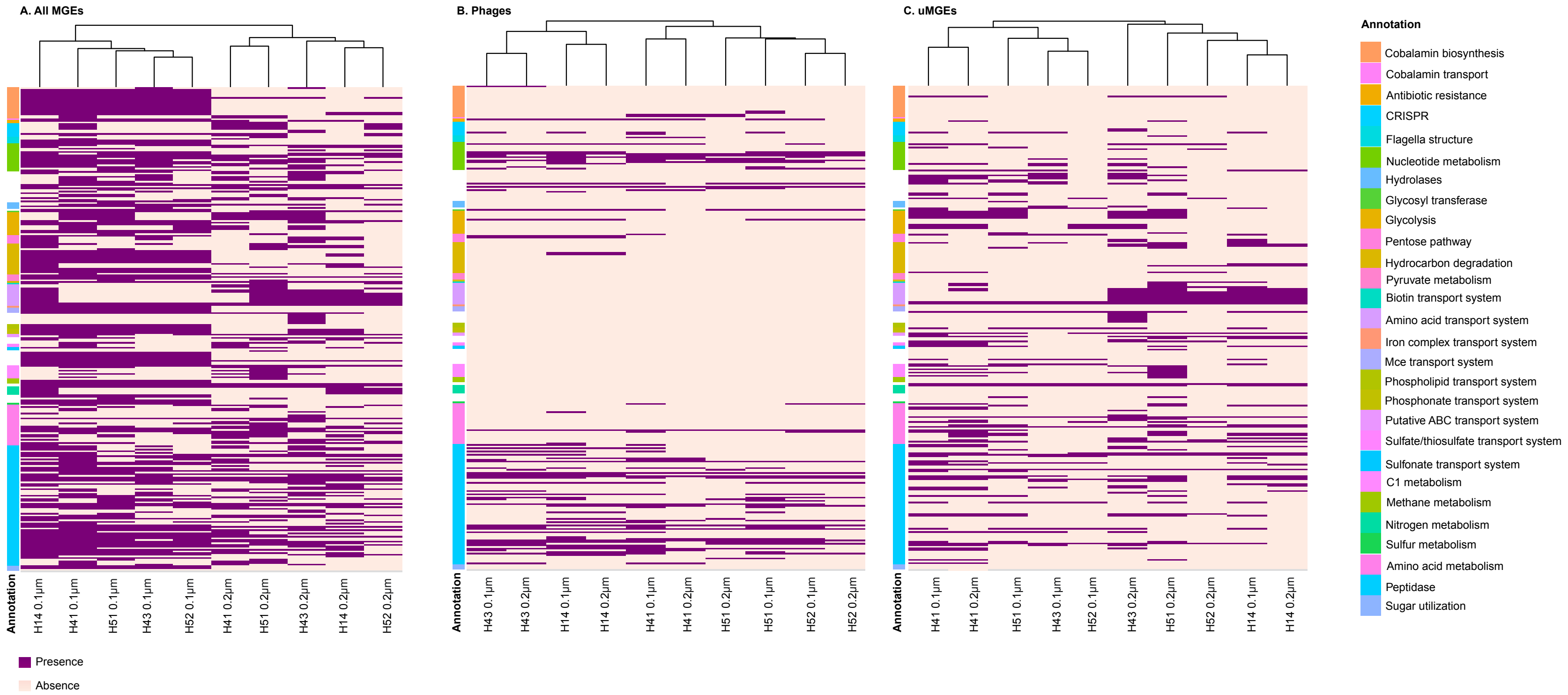

Supplementary Figure S10. Metabolic differences across sites and filter fractions in all the MGE (A), phage (B), and uMGE (C) sequences. The heatmap shows presence (purple squares) and absence (pink squares) of specific auxiliary metabolic genes (AMGs) in the MGE sequences across sites.

References

140. Darling, A. C., Mau, B., Blattner, F. R. & Perna, N. T. Mauve: multiple alignment of conserved genomic sequence with rearrangements. Genome Res. **14**, 1394–1403 (2004).
